# ESM2_AMP: an interpretable framework for protein–protein interactions prediction and biological mechanism discovery

**DOI:** 10.1093/bib/bbaf434

**Published:** 2025-08-28

**Authors:** Yawen Sun, Rui Wang, Zeyu Luo, Lejia Tan, Junhao Liu, Ruimeng Li, Dongqing Wei, Yu-Juan Zhang

**Affiliations:** College of Life Science, Chongqing Normal University, No. 37 University Town Road, high-tech District, Chongqing 401331, P.R. China; College of Life Science, Chongqing Normal University, No. 37 University Town Road, high-tech District, Chongqing 401331, P.R. China; College of Life Science, Chongqing Normal University, No. 37 University Town Road, high-tech District, Chongqing 401331, P.R. China; College of Life Science, Chongqing Normal University, No. 37 University Town Road, high-tech District, Chongqing 401331, P.R. China; College of Life Science, Chongqing Normal University, No. 37 University Town Road, high-tech District, Chongqing 401331, P.R. China; College of Life Science, Chongqing Normal University, No. 37 University Town Road, high-tech District, Chongqing 401331, P.R. China; State Key Laboratory of Microbial Metabolism, Joint International Research Laboratory of Metabolic & Developmental Sciences and School of Life Sciences and Biotechnology, Shanghai Jiao Tong University, 800 Dongchuan RD. Minhang District, Shanghai 200030, P.R. China; College of Life Science, Chongqing Normal University, No. 37 University Town Road, high-tech District, Chongqing 401331, P.R. China

**Keywords:** protein–protein interaction, interpretable analysis, attention mechanism, functional amino acid region

## Abstract

The prediction of binary protein–protein interactions (PPIs) is essential for protein engineering, but a major challenge in deep learning-based methods is the unknown decision-making process of the model. To address this challenge, we propose the ESM2_AMP framework, which utilizes the ESM2 protein language model for extracting segment features from actual amino acid sequences and integrates the Transformer model for feature fusion in binary PPIs prediction. Further, the two distinct models, ESM2_AMPS and ESM2_AMP_CSE are developed to systematically explore the contributions of segment features and combine with special tokens features in the decision-making process. The experimental results reveal that the model relying on segment features demonstrates strong correlations between segments with high attention weights and known functional regions of amino acid sequences. This insight suggests that attention to these segments helps capture biologically relevant functional and interaction-related information. By analyzing the coverage relationship between high-attention sequence fragments and functional regions, we validated the model’s ability to capture key segment features of PPIs and revealed the critical role of functional domains in PPIs. This finding not only enhances the interpretability methods for sequence-based prediction models but also provides biological evidence supporting the important regulatory role of functional sequences in protein–protein interactions. It offers cross-disciplinary insights for algorithm optimization and experimental validation research in the field of computational biology.

## Introduction

Protein–protein interactions (PPIs) are essential for understanding biomolecular functions, maintaining biological stability, and identifying target sites. These interactions are facilitated through non-covalent bonds, forming protein complexes crucial for cellular processes [1]. Studying PPI provides significant molecular biological insights, contributing to advancements in drug design and protein engineering [2–4]. Binary PPIs prediction is an important branch of PPIs research, focusing on predicting whether two proteins interact with each other [5]. Traditional experimental methods like yeast two-hybrid and Co-Immunoprecipitation, though effective, are labor-intensive, time-consuming, and costly [6, 7]. The growing demand for rapid identification of protein–protein interactions (protein binary interactions) has brought increasing attention to this field, spurring the development of innovative computational models.

The development of PPIs prediction has evolved through three key phases. Early machine learning approaches [8, 9] relied on handcrafted features, such as autocovariance and conjoint triad descriptors. The advent of deep learning introduced automated feature extraction through CNN (e.g., DPPI) [10], Siamese architectures (e.g., PIPR) [11] and embedding-based methods like Res2vec from DeepFE-PPI [12]. Recently, protein language models (e.g., ProtT5 [13], ESM2) have become dominant, with advanced architectures such as xCAPT5 [14] and TUnA [15] achieving superior performance by capturing rich contextual and sequential dependencies from raw amino acid sequences. However, most existing models lack interpretability analysis of their internal decision-making processes. A few studies, such as HIGH-PPI [16] identify binding sites to enhance interpretability. However, most studies do not thoroughly explore the internal logic of PPIs prediction models or incorporate classical attribution methods such as SHAP or Integrated Gradients (IG). Current trends aim to balance model performance and interpretability, while also reducing computational complexity to facilitate real-world biomedical applications of PPIs prediction. Protein language models (PLMs) have become the cutting-edge technology for representing protein sequences and predicting PPIs. PLMs, built on the Transformer architecture, can capture complex contextual information and evolutionary patterns within protein sequences. These models, particularly those based on the Transformer’s encoder-decoder framework [17], use the scaled dot-product attention mechanism to model relationships between amino acids, allowing the model to capture long-range dependencies and contextual information within sequences. For example, the ESM2 model, a variant of the BERT [18] architecture, treats amino acid sequences like text, encoding each amino acid by its type and position [19]. During pretraining, the model predicts masked residues, enabling it to learn meaningful sequence representations. The special [CLS] token at the beginning of a sequence aggregates contextual information, while the [EOS] token marks the end of the sequence. A more commonly used approach involves applying global pooling over amino acid sequences to obtain fixed-length representations. This approach allows the model to capture features that are essential for tasks such as classification or interaction prediction. Although global pooling and special token-based representations (e.g., [CLS], [EOS]) have been widely used for sequence-level prediction tasks (like protein binary interactions prediction), the function and interactions of proteins often depend on specific local regions, such as active sites or binding domains, which play critical roles in protein–protein and protein-ligand interactions [20]. Relying solely on global features and special token features for prediction may lead to the loss of crucial information regarding the interaction mechanisms and functional predictions, limiting the model’s ability to make accurate PPIs predictions.

Additionally, despite the significant progress made by PLMs in PPIs prediction, a key challenge remains deep learning models often produce decisions that are opaque to human researchers and may rely on inaccurate features and relationships for prediction. For instance, studies have revealed that PPIs prediction models depend on the intrinsic features of individual protein sequences, yet they have not thoroughly investigated whether these models can effectively capture the specific characteristics of PPIs [12, 21]. This issue can mislead the model with incorrect biological assumptions, compromising its generalization to real-world data. Therefore, understanding the model’s decision-making mechanisms is essential, not just for evaluating predictive performance but also for improving interpretability. Although attention mechanisms provide a degree of interpretability by highlighting features relevant to decision-making, existing methods still face limitations. For instance, the HIGH-PPI model [16] combines graph learning and attention to identify key binding and catalytic sites, while the TUnA model [15] leverages ESM2 features for PPIs prediction. However, these approaches struggle with (1) potential misalignment between attention-highlighted regions and biologically significant sites, (2) high computational demands for integrating residue features with attention matrices, and (3) limited ability to distinguish the contributions of different features, making it difficult to fully explain model decisions.

This study aims to address two key challenges in binary protein–protein interactions (PPIs) prediction: (1) enhancing the interpretability of feature representations while maintaining model performance without significantly increasing computational cost, and (2) uncovering the underlying mechanisms captured by ESM2 feature representations to link model decisions to biologically with biologically meaningful protein segments. To facilitate these purposes, we propose an interpretable framework ESM2_AMP, which leverages ESM2 to represent protein pairs at both the global pooling level, special token level represent Global representations and segment level represent through a dual-level feature extraction strategy. Global feature representations are derived from the mean pooling characteristics of the full-length sequence of amino acids, labeled as ‘ESM2_mean’. Special token feature representations extracted from [CLS] and [EOS] tokens, and that segment feature representations are obtained by segmenting the amino acid sequence into ten equal parts. The former are labeled as ‘ESM2_cls’ and ‘ESM2_eos’. Each segment representations are labeled as ‘ESM2_segment0–9’. These two types feature (exclude ESM2_mean) are fused using the multi-head attention mechanism, and a multilayer perceptron (MLP) [22] is employed for PPIs prediction. Additionally, two models, ESM2_AMPS and ESM2_AMP_CSE are constructed to investigate the impact of special token and segment features on prediction performance. Our method achieved high accuracy on the standard human dataset (Pan_dataset) [23] and was successfully extended to multi-species scenarios (Multi_species_dataset) [11]. Additionally, it was rigorously evaluated on the gold-standard Bernett dataset [24], designed to prevent data leakage, further demonstrating its robustness.

To enhance model interpretability and uncover the underlying mechanisms of protein–protein interaction, the ESM2_AMP framework introduces an innovative segment-based representation approach. This approach also facilitates the discovery of potential protein–protein interaction mechanisms, which offers clear advantages over traditional residue-level encodings derived from PLMs for Attention modeling, as the segment-based pooling dramatically reduces the number of tokens to be processed, thereby improving computational efficiency without sacrificing interpretability. Notably, the segment-level representations enable more efficient and biologically meaningful analysis of interaction patterns, offering clear advantages over existing global pooling strategies. Considering interpretation technic may differ in interpretation outcome [25], the SHAP and Integrated Gradient (IG) methods are further incorporate as comparison with the attention mechanism [26], achieving multi-level and multi-dimensional model interpretation. By leveraging an advanced importance quantification method, this study identifies critical segment features that drive prediction outcomes, directly linking the model’s decisions to known protein functional amino acid regions. In summary, ESM2_AMP leverages its robust interpretability capabilities to provide mechanistic insights into protein–protein interaction prediction and holds great potential for applications in drug discovery and functional genomics.

## Methods

### Protein–protein interaction datasets

Human protein interaction data were obtained from the Pan_dataset [23], with protein samples filtered to include sequences between 15–4000 amino acids in length. We constructed the training set through random 1:1 sampling of protein interaction pairs, resulting in 36,092 positive and 36,092 negative pairs (72,184 total) for model training. A five-fold cross-validation scheme was adopted, where 80% of the data was used for training and 20% for validation in each fold. For test, the real_test dataset was curated using Zhao et al.’s framework [27] combined with the Negatome database [28], containing 1200 positive and 1207 negative samples. To further ensure data independence, proteins in the real_test dataset exhibiting high sequence similarity (> 50% identity) with those in the Pan_dataset were systematically removed during use NCBI Blast.

The Bernett dataset, derived from human gold-standard interactions [24], underwent rigorous partitioning to prevent data leakage. In constructing this dataset, Bernett et al. implemented a rigorous multi-step procedure to strictly partition the data into training, validation, and test sets to prevent data leakage. The partitioning employed the KaHIP [29] strategy rather than relying solely on sequence similarity, ensuring no overlap between the training, validation, and test sets while minimizing sequence similarity across them. Additionally, CD-HIT was used to eliminate redundancy by removing proteins with high pairwise sequence similarity within each set [24]. Following removal of proteins exceeding 4000 amino acids, the final dataset contained 20,303 unique proteins distributed across three subsets: Training set: 79,961 positive and 79,983 negative interactions, Validation set: 29,334 positive and 29,327 negative interactions, Test set: 25,957 positive and 25,956 negative interactions. To assess cross-species generalization, incorporated the Multi_species_dataset from Chen et al. [11] (Table 1).

**Table 1:** Datasets information.

Dataset name	Species	Total proteins	Positive samples	Negative samples
Pan_dataset	Human	9619	36,092	36,092
real_test	Human	1741	1200	1207
Multi_species_dataset	Multiple species^*^	11,508	32,885	32,848
Bernett dataset	Human	20,303	Train: 79961 Val: 29334 Test: 25957	Train: 79983 Val: 29327 Test: 25956

Multiple species^*^ refers to the three species: *C. elegans*, *D. melanogaster*, and *E. coli*.

### Overview of ESM2_AMP framework

**Figure 1:**
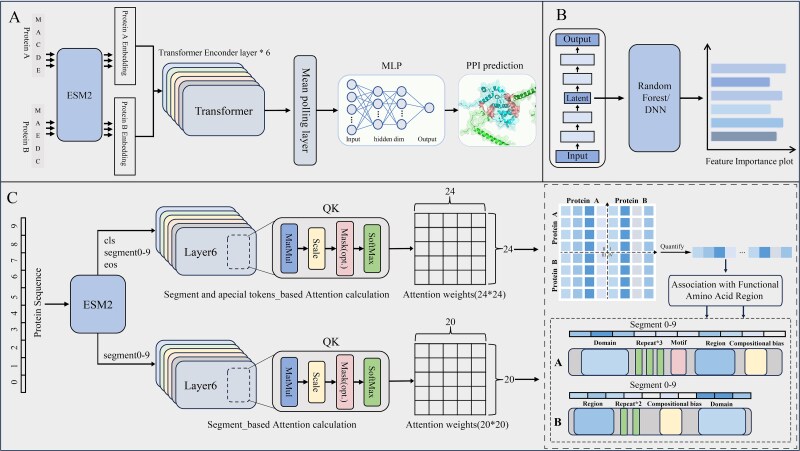
The ESM2_AMP framework and its two interpretability methods. (A) ESM2_AMP architecture. (B) Feature attribution module. (C) Attention mechanism interpretability module.

Based on attention feature fusion methods, we propose ESM2_AMP, an interpretable framework for predicting binary protein–protein interactions (PPIs). The framework initiates the feature extraction process by utilizing the pretrained protein language model ESM2 to generate comprehensive feature embeddings for each input protein sequence. These features are subsequently processed by a Transformer encoder, which utilizes a self-attention mechanism to integrate features from both proteins, effectively capturing both intra-protein interaction relationships and inter-protein interaction patterns. Finally, the integrated features are passed to a multilayer perceptron (MLP) classifier to predict whether a protein pair interacts (Fig. 1A).

### Feature extraction and fusion method

In ESM_AMP framework, ESM2, a Transformer-based protein language model, are deployed to extract features (ESM2_cls, ESM2_eos, ESM2_mean) from protein sequences. Specifically, ESM2_cls represents the CLS identifier (referred to as BOS in the original ESM2 paper [19]) located at the start of the sequence, ESM2_eos represents the EOS identifier positioned at the end of the sequence. ESM2 model specifically uses the ESM2_650M version. Inspired by Protloc_mex_X [30], the sequence is divided into ten segments, and segment features (ESM2_segment0–9) are obtained through segment residue sequence average pooling. For instance, ESM2_segment0_mean (abbreviated as ESM2_segment0) is one such segment feature. Details are in formula (1):


(1) \begin{align*} \left(S,R\right)&= divmod\left(L,N\right)\nonumber\\{}{E}_i&=\left\{\!\!\begin{array}{c}{E}_{i-1}+S+1\ if\ i<R,\\{}{E}_{i-1}+S\ otherwise\end{array}\right.\nonumber\\[4pt] {}{Sub}_i&=\left\{\!\!\begin{array}{c}H\left[{E}_{i-1}:{E}_i\right]\ if\ {E}_i>{E}_{i-1},\nonumber\\{}\overrightarrow{0}\kern0.5em otherwise\ \end{array}\right.\\{} Specifically,\,{E}_{i-1}&=0\ if\ i=0. \end{align*}

Here, L represents the length of the amino acid sequence, N is the number of segments (set to 10), S denotes the length of each segment, R is the remainder, ${E}_i$ indicates the end position of segment $i$, H represents the features of the last hidden layer of the sequence, and ${Sub}_i$ refers to the features of all residues within segment $i$. The average feature of each segment is calculated through average pooling, where $i$ corresponds to the segment index ($i=0$ represents the first segment, starting at position 0 of the sequence).

Using this method, a total of 13 representation features are obtained for a single protein sequence, each with 1280 dimensions. Subsequently, these features are used as representations of individual proteins for further research in protein–protein interactions.

Transformer and Multi-head Attention Mechanism.

The Transformer architecture [17], served as the adaptive model for fine-tuning ESM2 model for binary PPIs prediction. The architecture consists of six encoder layers, each comprising N identical layers. The encoder layers are built with a multi-head self-attention module and a feed-forward neural network. Details are in formula (2):


(2) \begin{align*} \mathrm{MultiHead}\ \left(\mathrm{Q},\mathrm{K},\mathrm{V}\right)&=\mathrm{Concat}\left({head}_1,{head}_2,\dots, {head}_h\right){W}_O\nonumber\\{}{head}_i&=\mathrm{Attention}\left(Q{W_i}^Q,K{W_i}^K,V{W_i}^V\right)\nonumber\\{}\mathrm{Attention}\ \left(\mathrm{Q},\mathrm{K},\mathrm{V}\right)&=\mathrm{softmax}\left(\frac{QK^T}{\sqrt{d_k}}\right)V \end{align*}

${W_i}^Q$ representsthe query projection matrix for the $i- th$ attention head, where $Q$ is the query vector, and $Q{W_i}^Q$ is the transformed query vector obtained by applying the query projection matrix ${W_i}^Q$ to the input sequence $X$. ${W_i}^K$ denotes the key projection matrix for the $i- th$ attention head, where K is the key vector, and $K{W_i}^K$ is the transformed key vector obtained by applying the key projection matrix ${W_i}^K$ to the input sequence $X$. ${W_i}^V$ signifies the value projection matrix for the $i- th$ attention head, where $V$ is the value vector, and $V{W_i}^V$ is the transformed value vector obtained by applying the value projection matrix ${W_i}^V$ to the input sequence $X$.

### Construction of dual models based on feature integration

Based on different feature integration methods and the basic ESM2_AMP framework, the ESM2_AMPS and ESM2_AMP_CSE models were developed.

The ESM2_AMPS model extracted segment features for each protein pair, labeled as ‘A_segment0–9’ and ‘B_segment0–9’. These features are concatenated into a matrix $\mathrm{E}\in{\mathbb{R}}^{20\times 1280}$ and input into Transformer encoder. The self-attention mechanism integrates these features, capturing intra-protein and inter-protein interaction patterns. The ESM2_AMP_CSE model combined segment features (ESM2_segment0–9) with special tokens features (ESM2_cls and ESM2_eos). Features are concatenated in the order: A_cls, A_segment0–9, A_eos, followed by B_cls, B_segment0–9, B_eos. Additionally, a contrast model (ESM2_DPM) uses only global features (ESM2_mean) from proteins A and B. These features are fed into a deep neural network (DNN) [31, 32] to predict PPIs, serving as a baseline to evaluate the impact of segment versus special token features.

Collectively, these models aim to explore the roles of segment versus special token features in binary PPIs prediction, with ESM2_AMPS focusing on interactions and ESM2_AMP_CSE integrating both actual segment sequence and tokens’ information. ESM2_DPM as a baseline for evaluating the performance of global features alone.

To investigate the significance of the attention mechanism in the Transformer architecture, an ablation experiment was conducted by removing the Transformer encoder module from the ESM2_AMPS model. To investigate the importance of the attention mechanism in Transformer architecture, an ablation experiment was conducted by removing the Transformer encoder module from the ESM2_AMPS model. The resulting model was named ESM2_mean_MPS. To investigate the impact of different protein language models on feature extraction for prediction and the necessity of selecting ESM2 as the feature extractor for protein features, the ProtT5 protein language model was chosen to replace ESM2 for obtaining protein representations, while keeping other modules unchanged. The resulting model was named ProtT5_AMPS. ProtT5 is based on the T5 model from the field of natural language processing, combined with protein sequence information, and is used for protein-related tasks. The version used in this study is ProtT5-XL-UniRef50, which produces feature’s token with a dimensionality of 1024.

Additionally, the model training integrated deep learning optimization techniques to achieve both computational efficiency and good predictive performance. The architecture employed the AdamW optimizer [33], which enhances regularization stability by decoupling weight decay from gradient updates. Hyperparameter optimization was automated through Optuna [34], conducting systematic searches across critical parameters: initial learning rate, weight decay coefficient and hidden layer dimensionality. The network architecture incorporated ReLU activation functions [35] to ensure robust gradient flow and prevent vanishing gradients, while Kaiming initialization [36] maintained stable activation distributions across all layers.

### Methodology for model training and performance evaluation

Model performance was evaluated using metrics such as Accuracy and MCC [37], with specific evaluation metrics and calculation methods shown in the following formula (3):


(3) \begin{align*} Accuracy&=\frac{TP+ TN}{TP+ TN+ FP+ FN}\nonumber\\{} Recall&=\frac{TP}{TP+ FN}\nonumber\\{} Precision&=\frac{TP}{TP+ FP}\nonumber\\{}F1&=2\times \frac{Precision\times Recall}{Precision+ Recall}\nonumber\\{} MCC&=\frac{TP\times TN- FP\times FN}{\sqrt{\left( TP+ FP\right)\left( TP+ FN\right)\left( TN+ FP\right)\left( TN+ FN\right)}} \end{align*}

TP (True Positive) is the count of actual positive samples correctly identified as positive, and TN (True Negative) is the count of actual negative samples correctly identified as negative. FP (False Positive) counts actual negative samples wrongly labeled as positive; FN (False Negative) counts actual positive samples wrongly labeled as negative. Accuracy measures the ratio of correct predictions (TP + TN) to the total samples. Recall, or TPR (True Positive Rate), measures the ratio of TP to all actual positives (TP + FN). Precision measures the ratio of TP to all predicted positives (TP + FP). The F1 Score averages Precision and Recall harmonically. AUC is the area under the ROC curve plotting TPR versus FPR. MCC evaluates binary classification by considering TP, TN, FP, and FN, fitting for imbalanced datasets.

### Interpretability analysis utilizing attention mechanisms

To gain a deeper understanding of the decision-making mechanisms of the ESM2_AMPS and ESM2_AMP_CSE models in predicting binary PPIs, Attention weights were computed similarly and used for attention significant maps, allowing comparison of local and global feature importance. For ESM2_AMPS, segment features (A_segment0–9 and B_segment0–9) were combined into a 20 × 1280 matrix. The Transformer’s self-attention calculated weights, creating attention significant maps. ESM2_AMP_CSE added special token features (A_cls, A_eos, B_cls, B_eos), forming a 24 × 1280 matrix.

In Transformer architecture, Attention Weights are calculated:


(4) \begin{equation*} \mathrm{Attention}\ \mathrm{Weights}=\mathrm{softmax}\left(\frac{QK^T}{\sqrt{d_k}}\right) \end{equation*}

Basically, it’s just removed V weight metrics from attention formula (4).

### Autoencoder model construction and feature importance calculation methods

Autoencoder (AE) are an unsupervised deep learning algorithm consisting of an input layer, Encoder, hidden layer, Decoder, and output layer [38]. AE uses backpropagation to ensure the output matches the input data structure. The Encoder generates a latent space representation (embedding), and the Decoder reconstructs the high-dimensional data from this representation. During dimensionality reduction, AE retains key information while removing noise and redundant features.

In this research, the AE algorithm was used to reduce the dimensionality of protein pair features. For feature integration method 1, the 3D tensor $X\in{\mathbb{R}}^{2407\times 20\times 1280}$ from the real_test dataset was reduced to $Y\in{\mathbb{R}}^{2407\times 20\times 150}$. Each protein pair is represented as a 20 × 150 matrix $Z\in{\mathbb{R}}^{20\times 150}$, which is then flattened into a 1D vector with 3000 features. For feature integration method 2, the 3D tensor $M\in{\mathbb{R}}^{2407\times 24\times 1280}$ was reduced to $N\in{\mathbb{R}}^{2407\times 24\times 150}$. Each protein pair is represented as a 24 × 150 matrix $K\in{\mathbb{R}}^{24\times 150}$, which is then flattened into a 1D vector with 3600 features. The reduced-dimensionality features were then used as input for a downstream RF or DNN model.

Besides, Tree SHAP (SHapley Additive exPlanations) [39] is used as the comparison model interpretation methods for attention weight, dimensionality-reduced features from one Autoencoders (AE) [40] were input into an Random forest (RF) [41] model (AE_RF), and Tree SHAP was applied to calculate Shapley values. Additionally, the Gini importance [42] derived from the Gini index was calculated for the RF model to quantify feature importance. Meanwhile, building upon the unsupervised feature representation from the AE, a downstream classifier utilizing DNN was implemented and referred to as ‘AE_DNN’. To interpret feature importance, the IG and SHAP attribution methods were employed to analyze the model’s features. Notably, since each feature has a dimensionality of 150, the feature importance score for each feature is calculated by averaging the absolute values across all dimensions of the feature to represent its overall importance. This allowed analysis of feature importance under different integration methods, which was compared with attention mechanism-derived feature importance to better understand the model’s decision-making process.

### Identification and computational methods of functional amino acid regions

To investigate potential correlations between feature weights and specific residues or regions, the ESM2_AMPS model, which utilizes segment features, was analyzed. Using the real_test dataset, the top three features with the highest attention weights for each sample were identified, and the proportion of functional amino acid sequences [43–47] they covered was calculated. For comparison, the three features with the lowest weights were selected as a negative control group. This analysis aims to evaluate whether feature importance aligns with biologically significant regions in protein sequences.

First, the samples from the real_test dataset were classified into four categories based on their true and predicted labels: TP (True Positive), FP (False Positive), TN (True Negative), and FN (False Negative). For the proteins in these pairs, five functional amino acid sequence regions were extracted from the UniProt database [48]. The coverage of functional regions within each segment was then calculated, defined as the proportion of functional sequences within the segment’s corresponding region, as outlined in Formula (5):


(5) \begin{align*} {\overline{\boldsymbol{C}}}_{\boldsymbol{i}}^{\left(\boldsymbol{k}\right)}&=\frac{\mathbf{1}}{\boldsymbol{N}}\sum_{\boldsymbol{n}=\mathbf{1}}^{\boldsymbol{N}}\left(\frac{\sum_{\boldsymbol{j}}\mathrm{UniqueOverlap}\left({\boldsymbol{S}}_{\boldsymbol{k}\boldsymbol{jn}},{\boldsymbol{T}}_{\boldsymbol{i}\boldsymbol{n}}\right)}{{\boldsymbol{L}}_{\boldsymbol{i}\boldsymbol{n}}}\right)\times \mathbf{100}\%\nonumber\\{}{\overline{\boldsymbol{C}}}_{\boldsymbol{i}}^{\left(\mathrm{all}\right)}&=\frac{\mathbf{1}}{\boldsymbol{N}}\sum_{\boldsymbol{n}=\mathbf{1}}^{\boldsymbol{N}}\left(\frac{\sum_{\boldsymbol{k},\boldsymbol{j}}\mathrm{UniqueOverlap}\left({\boldsymbol{S}}_{\boldsymbol{k}\boldsymbol{jn}},{\boldsymbol{T}}_{\boldsymbol{i}\boldsymbol{n}}\right)}{{\boldsymbol{L}}_{\boldsymbol{i}\boldsymbol{n}}}\right)\times \mathbf{100}\%\nonumber\\{}{\overline{\boldsymbol{C}}}^{\left(\boldsymbol{k}\right)}&=\frac{\mathbf{1}}{\mathbf{3}\boldsymbol{N}}\sum_{\boldsymbol{n}=\mathbf{1}}^{\boldsymbol{N}}\sum_{\boldsymbol{i}=\mathbf{1}}^{\mathbf{3}}\left(\frac{\sum_{\boldsymbol{j}}\mathrm{UniqueOverlap}\left({\boldsymbol{S}}_{\boldsymbol{k}\boldsymbol{jn}},{\boldsymbol{T}}_{\boldsymbol{i}\boldsymbol{n}}\right)}{{\boldsymbol{L}}_{\boldsymbol{i}\boldsymbol{n}}}\right)\times \mathbf{100}\%\nonumber\\{}{\overline{\boldsymbol{C}}}^{\left(\mathrm{all}\right)}&=\frac{\mathbf{1}}{\mathbf{3}\boldsymbol{N}}\sum_{\boldsymbol{n}=\mathbf{1}}^{\boldsymbol{N}}\sum_{\boldsymbol{i}=\mathbf{1}}^{\mathbf{3}}\left(\frac{\sum_{\boldsymbol{k},\boldsymbol{j}}\mathrm{UniqueOverlap}\left({\boldsymbol{S}}_{\boldsymbol{k}\boldsymbol{jn}},{\boldsymbol{T}}_{\boldsymbol{i}\boldsymbol{n}}\right)}{{\boldsymbol{L}}_{\boldsymbol{i}\boldsymbol{n}}}\right)\times \mathbf{100}\% \end{align*}

$\boldsymbol{i}$ : Top fragment index, with values in the set, corresponding to Top1, Top2, Top3 (or Low1, Low2, Low3); $\boldsymbol{k}$: Functional type, with values in the set {Domain, Region, Compositional bias, Repeat, Motif}; ${\boldsymbol{S}}_{\boldsymbol{kjn}}$: The $\boldsymbol{j}$th interval of the $\boldsymbol{k}$th type special segment in the $n$th sample, represented as [start, end]; ${T}_{in}$: The $i$th Top (or Low) fragment in the $n$th sample, represented as (start, end]; ${L}_{in}$: Interval length of ${T}_{in}$ in the $n$th sample, i.e., (end - start); $\mathrm{Unique}\ \mathrm{Overlap}\left(A,B\right)$: Represents the unique overlap portion between intervals $A$ and $B$; $N$: Total number of samples; ${\overline{C}}_i^{(k)}$: Overall average coverage of a specific type of special segment corresponding to the $i$th Top (or Low); ${\overline{C}}_i^{\left(\mathrm{all}\right)}$: Overall average coverage of all types of special segments corresponding to the $i$th Top (or Low); ${\overline{C}}^{(k)}$: Overall average coverage of a specific type of special segment corresponding to three Top (or Low) across all samples; ${\overline{C}}^{\left(\mathrm{all}\right)}$: Overall average coverage of all special segments corresponding to three Top (or Low) across all samples.

Secondly, a threshold is set based on each segment’s sequence coverage. Coverage exceeding the threshold is marked a ‘hit’ (recorded as 1); otherwise, it is not a hit (recorded as 0). The hit rate for TP, FP, TN, and FN is calculated as the proportion of samples where the top three weighted segments hit the functional amino acid sequence regions, relative to the total samples in each category, as shown in Formula (6):


(6) \begin{align*} {I}_k^{(i)}(x)&=\left\{\!\!\begin{array}{ll}1,& x\ge \theta \\{}0,& x<\theta \end{array}\right.\nonumber\\{}{P}_k^{(i)}&=\left(\frac{\sum_{n=1}^N{I}_k^{(i)}\left({x}_n\right)}{N}\right)\times 100\%\nonumber\\{}{P}_k^{\left(\mathrm{all}\right)}&=\left(\frac{\sum_{n=1}^N\max \left({I}_k^{(1)}\left({x}_n\right),{I}_k^{(2)}\left({x}_n\right),{I}_k^{(3)}\left({x}_n\right)\right)}{N}\right)\times 100\%\nonumber\\{}{P}_{\mathrm{ALL}}^{(i)}&=\left(\frac{\sum_{n=1}^N\prod_k{I}_k^{(i)}\left({x}_n\right)}{N}\right)\times 100\% \end{align*}

$x$ : Coverage value, representing the coverage ratio of a certain Top fragment on a specific functional type; $\theta$: Preset threshold, with values of 25%, 50%, or 75%; ${I}_k^{(i)}(x)$: Indicator function, indicating whether the coverage of the $i$th Top fragment on functional type $k$ reaches the threshold $\theta$. If the coverage $x\ge \theta$, then ${I}_k^{(i)}(x)=1$; otherwise, it is $0$; ${x}_n$: The coverage value of the $n$th sample; $k$: Functional type, with values in the set $\left\{ Domain, Region, Compositional\ bias, Repeat, Motif\right\}$; $i$: Top fragment index, with values in the set $\left\{1,2,3\right\}$, corresponding to Top1, Top2, Top3 (or Low1, Low2, Low3); $N$: Total number of samples; ${P}_k^{(i)}$: Hit rate of functional type $k$ on the $i$th Top fragment; $\max \left(\cdotp \right)$: Maximum value function, used to determine if at least one of the three Top or Low fragments meets the condition; ${P}_k^{\left(\mathrm{all}\right)}$: Combined hit rate of functional type $k$ on at least one of the three Top or Low fragments (a total of three Top or Low fragments); $\prod_k\kern0em$: Product over all functional types $k$, used to determine if all functional types meet the conditions simultaneously; ${P}_{\mathrm{ALL}}^{(i)}$: Overall hit rate of all functional types on the $i$th Top or Low fragment.

In addition, coverage and hit rates were calculated for each of the five functional amino acid sequence regions, with calculations based on these specific functional sequence types.

This method assesses whether model-identified features concentrate in biologically important regions critical for PPIs. It validates model effectiveness and offers insights into protein interactions via specific regions or residues. Also, by comparing coverage and hit rate differences across TP, FP, TN, and FN, and analyzing attention weight differences for segments in TP, we reveal key features for PPIs prediction. This shows the model can learn biologically relevant info from local segments.

## Results

### Benchmarking evaluation for ESM2_AMP on PPIs prediction

**Figure 2:**
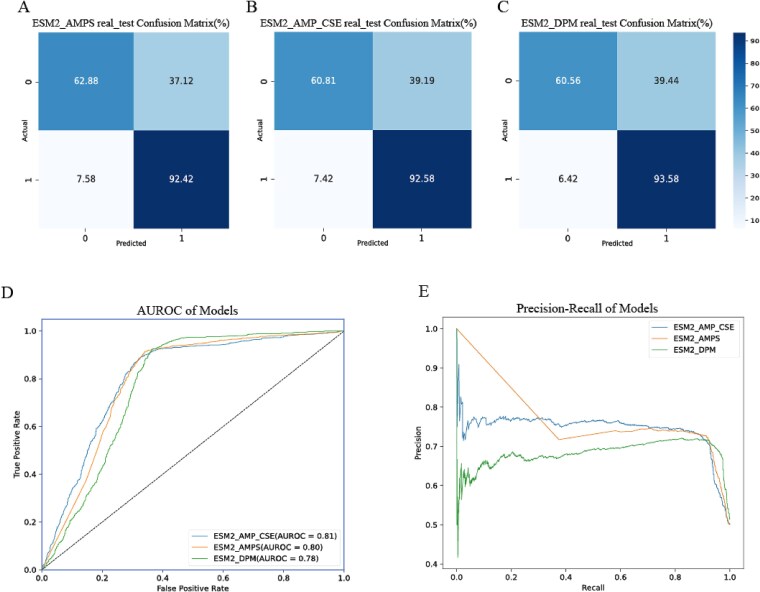
Evaluation of three models on the real_test dataset. A-C: Confusion matrices depicting actual (0 = non-interacting, 1 = interacting) versus predicted labels:(A) ESM2_AMPS, (B) ESM2_AMP_CSE, (C) ESM2_DPM. D: AUROC curves for the three models. E: Precision-recall curves for the three models.

During model training and validation, a systematic evaluation approach was implemented to comprehensively assess the performance of the three constructed binary protein–protein interaction prediction models (ESM2_AMPS, ESM2_AMP_CSE, and ESM2_DPM). For the validation phase, the human protein interaction benchmark dataset (Pan_dataset), containing experimentally validated high-quality protein interaction pairs, was selected. To ensure the reliability of the evaluation results, a five-fold cross-validation experimental design was adopted. The results demonstrated that all three models exhibited stable predictive performance across all validation folds (Table 2, Fig. 2D-E). Notably, the AUC achieved an excellent level, exceeding 0.99 for all models. Simultaneously, accuracy remained consistently high, above 0.97, further validating the precision of these model predictions.

**Table 2:** Five-fold cross-validation performances of different models on Pan_dataset.

	Accuracy	Recall	F1 Score	AUC	MCC
ESM2_AMPS	0.9776	0.9708	0.9775	0.9949	0.9553
ESM2_AMP_CSE	0.9787	0.974	0.9786	0.9937	0.9574
ESM2_DPM	0.9802	0.9738	0.9801	0.9959	0.9605

The results demonstrate the prediction performance of the three models on real_test dataset (Table 3), with the ESM2_AMPS model exhibiting slightly superior performance compared to the other two models in terms of accuracy, F1 score, MCC, and precision, which suggests that segment feature sampling within a local range may capture more information relevant to the PPIs process than special tokens and traditional pooling features, thereby enhancing model prediction performance. The Precision-Recall curve of the ESM2_AMPS model differs from the fluctuations of the other two models. At the beginning, the precision is relatively high. As the recall increases, the precision drops rapidly, and after the recall reaches a certain value (about 0.4), the downward trend slows down. The reason for the smaller fluctuation may be that the positive and negative sets of the segmented features used by the ESM2_AMPS model are evenly distributed, without extreme situations of some features being dense or sparse. Therefore, when the model adjusts the decision-making threshold to change the recall, there will be no significant fluctuations in prediction results due to the influence of some features.

**Table 3:** Model prediction performances on real_test dataset.

	Accuracy	Recall	F1 Score	AUC	MCC	Precision
ESM2_AMPS	0.7761	0.9242	0.8045	0.7932	0.5785	0.7123
ESM2_AMP_CSE	0.7665	0.9258	0.7981	0.8064	0.5628	0.7014
ESM2_DPM	0.7703	0.9358	0.8024	0.7767	0.5733	0.7023

Furthermore, in the real_test dataset predictions of the three models, the probability of correctly predicting positive samples reached as high as 90%, while the probability of correctly identifying negative samples was only around 60%. (Fig. 2A-C). This discrepancy may be related to the distribution disparity of the train dataset and real_test dataset. The real_test dataset samples and the positive samples in the train dataset (Pan_dataset) are both derived from real experimental data [28], the negative samples in the train dataset is generated based on the different subcellular compartments where the proteins are located [23]. For performance evaluation, use the real_test dataset after removing proteins with high sequence similarity as test set, named ‘de-homology real_test dataset’. Comparative analysis revealed distinct prediction patterns between the filtered dataset and the original dataset: the prediction accuracy for negative samples increased, while the accuracy for positive samples decreased after removing high-similarity sequences. Among the three models, ESM2_AMPS demonstrated the highest true negative rate (76.8%), outperforming both ESM2_AMP_CSE (75%) and ESM2_DPM (66.1%). Conversely, for true positive rate, ESM2_DPM showed superior performance compared to ESM2_AMPS, which in turn outperformed ESM2_AMP_CSE (Fig. S1A-C).

The evaluation incorporated an additional test set, named the ‘de-homology Bernett test set’, constructed by removing protein pairs with high sequence similarity between the Bernett dataset test set and the Pan_dataset. This rigorous filtering approach yielded several notable observations: (1) prediction accuracy remained substantially higher for positive samples compared to negative samples, and (2) model performance exhibited limited generalizability, potentially attributable to the exclusion of biologically relevant yet sequence-distant interactions through the stringent filtering criteria (Fig. S1D). The above results indicate that segment features sampling may capture protein–protein interaction related information more effectively than special tokens and traditional pooling features, thereby enhancing predictive performance.

**Figure 3:**
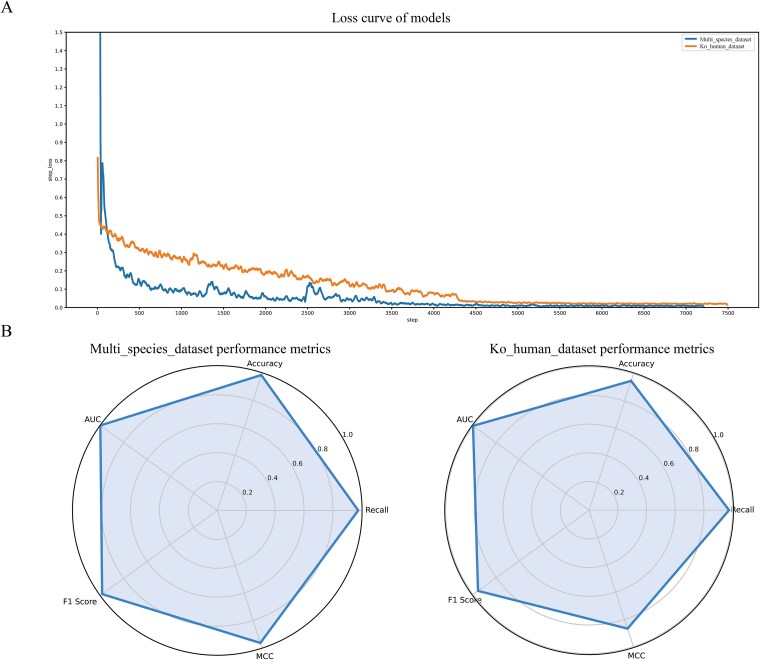
Performance of the datasets on ESM2_AMPS model using five-fold cross-validation. (A) Multi_species_dataset and Ko_human_dataset validation loss curve. (B) Multi_species_dataset and Ko_human_dataset validation set performance radar chart.

The research investigated the generalization capability of the ESM2_AMPS model, which relies solely on segment features, across Multi_species_dataset and Ko_human_dataset. The results (Fig. 3) demonstrate that the model’s loss function value exhibited a stable decreasing trend and eventually converged during training. This training dynamic curve convincingly proves that the ESM2_AMPS model can effectively learn common feature patterns of protein–protein interactions. Radar charts were employed to visualize the model’s performance across multiple evaluation metrics on the datasets, showing that the ESM2_AMPS model performs well on the data of different sources. The ESM2_AMPS model demonstrated excellent generalization capabilities on the two datasets, indicating its strong potential in more scenarios.

### Contrastive analysis: Performance benchmarking of models on strictly constructed dataset

To mitigate potential data leakage between the training and test sets and to avoid overestimating model performance, we adopted the Bernett dataset proposed by Bernett et al as extension evaluation. This dataset is specifically designed with strict partitions for training, validation, and test sets, ensuring reliable and fair evaluations. It has been extensively used in validation across multiple sequence-based PPIs prediction models, making the evaluation results more comparable and trustworthy [24]. The performance data for thirteen models (Table 4) were provided by Young et al. [15] and Bernett et al [24].

**Table 4:** Performance on the Bernett dataset.

Model	AUROC	AUPR	Balanced Acc	Precision	Recall	Specificity	F1	MCC
DeepFE	0.52	0.51	0.52	0.52	0.47	0.57	0.49	0.04
DeepFE ES	0.53	0.53	0.50	0.00	0.00	1.00	0.00	/
PIPR	0.53	0.53	0.52	0.52	0.46	0.58	0.49	0.04
PIPR ES	0.54	0.53	0.52	0.52	0.67	0.37	0.58	0.04
D-SCRIPT	0.50	0.51	0.50	0.51	0.19	0.81	0.28	0.01
D-SCRIPT ES	0.56	0.58	0.55	0.76	0.15	0.95	0.25	0.17
Topsy-Turvy	0.59	0.59	0.56	0.65	0.26	0.86	0.37	0.15
Topsy-Turvy ES	0.60	0.62	0.53	0.80	0.08	0.98	0.15	0.14
ESM2-MLP	0.70	0.68	0.64	0.64	0.64	0.63	0.64	0.28
ESM2-GP	0.69	0.68	0.63	0.64	0.60	0.66	0.62	0.26
T-FC	0.69	0.68	0.63	0.62	0.69	0.57	0.65	0.27
TUnA-ProSE	0.70	0.68	0.65	0.65	0.64	0.65	0.65	0.29
TUnA	0.70	0.69	0.65	0.65	0.65	0.65	0.65	0.30
ESM2_AMPS	0.68	0.67	0.62	0.61	0.69	0.56	0.65	0.25
ESM2_GRU	0.69	0.68	0.64	0.62	0.69	0.58	0.66	0.27

We evaluated the ESM2_AMPS model on this dataset alongside its variant, ESM2_GRU, which replaces the Transformer module with a Gate Recurrent Unit (GRU) [49] module to better capture short-range dependencies. Both models exhibited superior performance in terms of recall and F1 scores compared to other models (Table 4), with most metrics outperforming non-PLM-based approaches, such as DeepFE [12], PIPR [11], D-SCRIPT [50], and Topsy-Turvy [51]. When compared to other PLM-based models [15], including ESM2-MLP, ESM2-GP, T-FC, TUnA-ProSE, and TUnA, the proposed models show only a slight performance difference (Table 4).

ESM2_GRU demonstrated marginally superior performance compared to ESM2_AMPS (with a 0.01–0.02 advantage), but the GRU model lacks interpretability of feature interactions. In contrast, ESM2_AMPS achieves a balance between performance and interpretability. Our results imply that complex Transformer architectures and attention mechanisms are not essential for constructing downstream fine-tuning models to enhance predictive performance. Additionally, this result emphasizes that the significance of using Transformer adapter models for interpretability research outweighs the focus on performance improvement. Specifically, it highlights the importance of further understanding the model’s underlying prediction mechanisms and uncovering biological mechanism knowledge embedded in model.

### Ablation experiments and comparison with other LLM features

**Figure 4:**
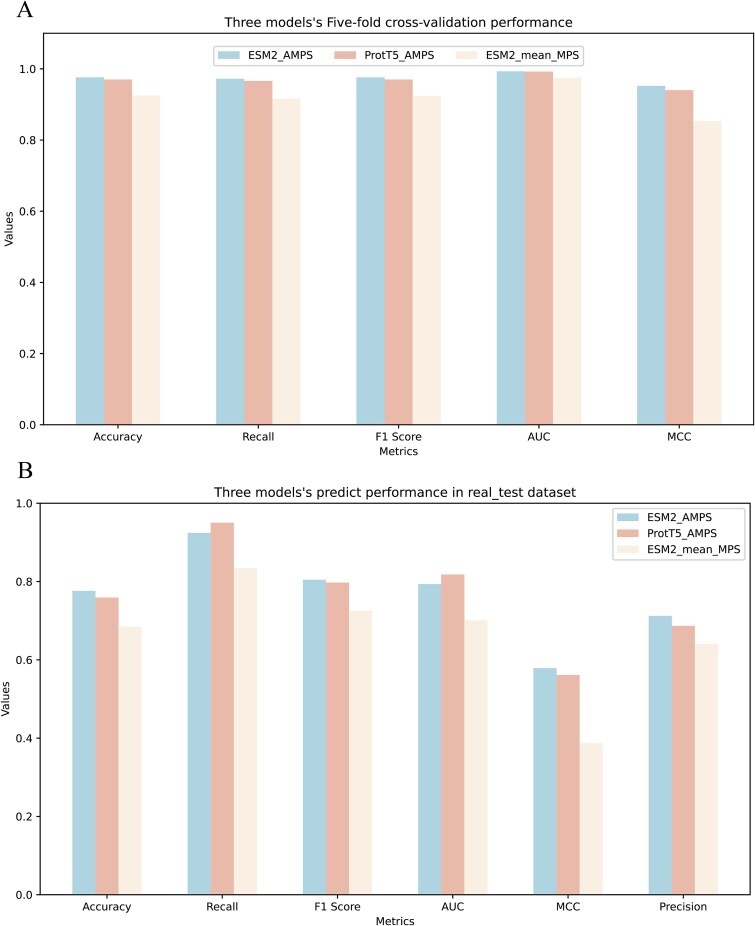
Five-fold cross-validation and predict performance of models. (A) Five-fold cross-validation performance of models (ESM2_AMPS, ProtT5_AMPS and ESM2_mean_MPS). (B) Predict performance of models (ESM2_AMPS, ProtT5_AMPS and ESM2_mean_MPS).

To validate the importance of the attention mechanism in the Transformer encoder for feature interaction and the superiority of ESM2 in feature extraction, we conducted ablation and comparative experiments based on the ESM2_AMPS model. Specifically, the ESM2_mean_MPS ablation model was created by removing the Transformer encoder, while the ProtT5_AMPS comparative model replaced ESM2 feature extraction with ProtT5 (Fig. 4).

Five-fold cross-validation on the Pan_dataset (Fig. 4A) demonstrated that ESM2_mean_MPS underperformed ESM2_AMPS across metrics, with a notable decrease in MCC (~0.1 lower). Independent testing on the real_test dataset further confirmed this degradation, yielding an MCC of only ~0.39 (Fig. 4B). Similarly, ProtT5_AMPS showed inferior performance to ESM2_AMPS in most metrics during cross-validation, though it achieved marginally higher Recall and AUC (Fig. 4A). These results highlight the critical role of the Transformer encoder’s attention mechanism and underscore ESM2 advantage over ProtT5 for feature extraction in this task.

### Attention mechanism reveal protein–protein interaction mechanism

To investigate whether PPIs model predictions are based on individual protein sequences or protein–protein interactions, the attention weights from the Self-Attention module in the ESM2_AMPS and ESM2_AMP_CSE models were analyzed. In ESM2_AMPS, the input features represent the segment features of protein pairs (‘A_segment0–9’ and ‘B_segment0–9’). In ESM2_AMP_CSE, special token features (‘A_cls’, ‘B_cls’) and ending embeddings (‘A_eos’, ‘B_eos’) are incorporated into the segment feature sequences.

The attention weights on the real_test dataset revealed no strong self-correlation among features, as the values on the main diagonal were not significantly higher than those between different features. This makes the interpretation of attention maps challenging. However, column-wise clustering of attention weights was observed in layers 2–6, indicating that column vectors in the attention weight matrix exhibited similar numerical values. This phenomenon was investigated at both integral and individual levels.

**Figure 5:**
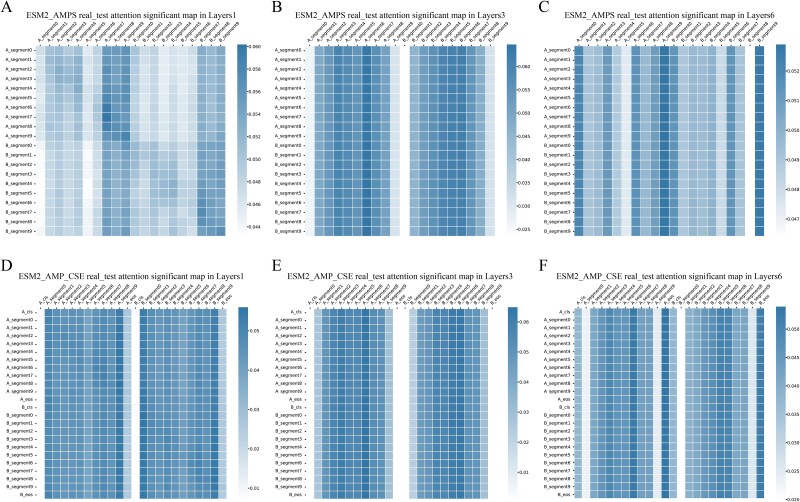
The average attention significant maps of protein pairs on real_test dataset. (A-C) attention significant maps of layers 1, 3, and 6 in the ESM2_AMPS model on real_test dataset. (D-F) attention significant maps of layers 1, 3, and 6 in the ESM2_AMP_CSE model on real_test dataset.

In the results of average total samples (integral level), the generalization and integration effects of the attention mechanism across protein samples were examined. In ESM2_AMPS, higher attention weights were observed for end segments (A_segment7–9 and B_segment7–9) in the first Transformer encoder layer, and weights for both ends segments (A_segment0, A_segment9, B_segment0, B_segment9) decreased in layers 2–5 and increased in the final layer (Fig. 5A-C, Fig. S2 A-C). Previous studies have demonstrated that Transformers can capture syntactic information between tokens. Similarly, amino acid sequences also exhibit analogous grammatical structures. The observed regular variations across different layers imply the existence of certain grammatical information related to the mechanisms of PPIs. This observation is consistent with previous findings in NLP research regarding the distinct focuses across different encoder layers in Transformer architecture [52]. In ESM2_AMP_CSE, the ‘cls’ and ‘eos’ features exhibited low attention weights in shallow layers but showed a significant increase in deeper layers (Fig. 5D-F, Fig. S2 D-F). This supports the hypothesis that shallow layers focus on local information; deeper layers emphasize global context. The use of residual connections and normalization enables the model to retain shallow-layer information and learning new features in deeper layers, aligning with previous findings [53]. Additionally, in the experiments, the average attention significant maps across all samples of the Bernett dataset in the ESM2_AMPS model demonstrated an overall distribution (Fig. S3) of attention weights like that observed in the real_test dataset.

**Figure 6:**
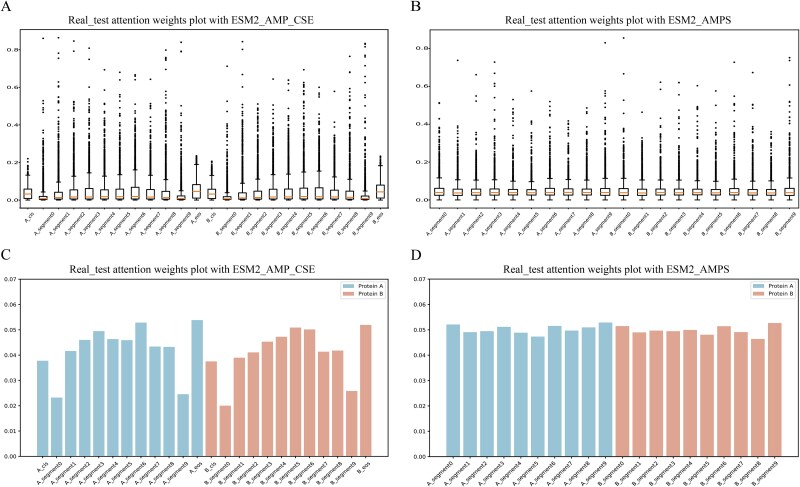
Attention value distribution and mean statistics. (A) Boxplot of average attention weights in the sixth layer of the ESM2_AMP_CSE model on the real_test dataset. (B) Boxplot of average attention weights in the sixth layer of the ESM2_AMPS model on the real_test dataset. (C) Bar chart of mean value average attention weights in the sixth layer of the ESM2_AMP_CSE model on the real_test dataset. (D) Bar chart of mean value average attention weights in the sixth layer of the ESM2_AMPS model on the real_test dataset.

Quantitative analysis of the real_test dataset using boxplots and bar plots revealed distinct attention patterns. In ESM2_AMPS model, shallow layers (layer 1) showed high variability in attention weights, with segment features (A_segment0, A_segment9, B_segment0, B_segment9) becoming more concentrated in layers 2–4 (narrower interquartile ranges). By layers 5–6, median values across features were nearly identical (maximum difference ~ 0.0028) (Fig. 6B, Fig. S5). Mean values for segment features increased from layers 2–5, converging in layer 6 with minimal differences (<0.007) (Fig. 6D, Fig. S7). In contrast, in ESM2_AMP_CSE model, special token features (A_cls, A_eos, B_cls, B_eos) had narrower distributions in shallow layers (1–3), which widened in deeper layers (5–6). Special token features exhibited smaller, more concentrated outliers, while local features had larger, more dispersed outliers (Fig. 6A, Fig. S6). Mean values for special token features increased in layers 2–4, surpassing most segment features by layers 5–6 (Fig. 6C, Fig. S8). Both quantitative analyses (boxplots and bar plots) consistently demonstrated, at the full-sample level, similar attention weight distributions for features of protein A and protein B across all layers.

This analysis (integral level) revealed distinct mechanisms of segment and special token feature learning between the two models. ESM2_AMPS tended to equalize the importance of all features in deeper layers, ESM2_AMP_CSE displayed a strong preference for special token features, potentially interfering with the significance of segment features. This suggests that special token features (‘cls’ and ‘eos’) may act as ‘sentence delimiters’ [18], potentially disrupting connections between segments of protein pairs and impairing the model’s ability to capture interactions (as evidenced by the performance drop on the independent test set after incorporating ‘cls’ and ‘eos’). These differences emphasize fundamental distinctions in feature integration strategies, specifically the contrast between combining special tokens and segment features and focusing solely on segment-specific features, providing valuable insights into feature selection mechanisms for PPIs prediction. Additionally, though intermediate layers exhibited similar attention weight distributions, the first and last layers displayed distinct patterns, reflecting the hierarchical and complex embedding mechanisms underlying PPIs.

A comparative experiment was conducted on single-sample (individual level) attention patterns and feature selection impacts. The attention weights of the ESM2_AMPS and ESM2_AMP_CSE models were analyzed for the protein pair ‘O14786-P08648’. This specific protein pair was chosen due to its presence in both the real_test dataset and the UniProt database, along with experimentally documented interaction.

**Figure 7:**
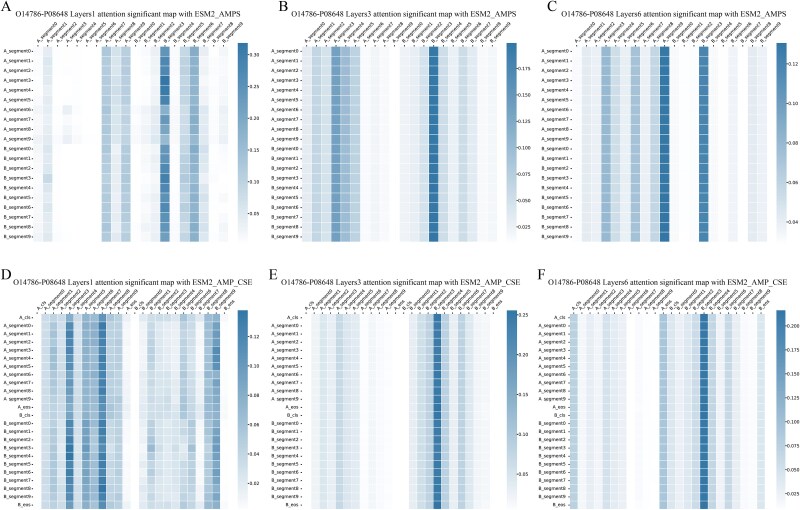
The layer-wise attention significant maps of ‘O14786-P08648’ protein pair in different models. (A-C) layer-wise attention significance maps (layers 1/3/6) for protein pair O14786-P08648 in ESM2_AMPS. (D-F) layer-wise attention significance maps (layers 1/3/6) for protein pair O14786-P08648 in ESM2_AMP_CSE.

In ESM2_AMPS model, feature B_segment3 had highest attention weights in layer 1, and A_segment3, A_segment6, A_segment9, and B_segment3 maintained higher attention weights in deeper layers (2–6), explained these segment features contain key functional information for protein interactions (Fig. 7A-C, Fig. S4 A-C). ESM2_AMP_CSE showed a different pattern: while B_segment3 also had high weights in deeper layers, unique global features (‘cls’ and ‘eos’) increased from shallow to deep layers, surpassing most segment features in the final layer, indicating special token features’ influence in integration (Fig. 7D-F, Fig. S4 D-F). The attention maps for the same segment features differed between models, and combined with performance results, suggest special token features distracted segment feature attention weight allocation.

To validate the generalizability of these findings, attention weight distributions were analyzed across multiple samples in the real_test dataset. For ESM2_AMPS, results (Fig. S11) showed consistent activation of segment features in deeper layers (2–6), with each sample having one or some segment features that consistently maintained high average attention weights. For ESM2_AMP_CSE, certain segment features also maintained relatively high average attention weights in deeper layers, but the increasing trend of special token feature attention weights was evident across multiple samples (Fig. S12).

Compared to integral level results, single sample (individual level) analysis directly uncovers how attention weights operate at the granular level, demonstrating that specific segment sequence segments decisively drive interaction predictions. These findings emphasized the unique interpretative power of individual samples in deciphering Transformer-based models, offering novel insights into feature integration mechanisms for protein interaction prediction. Importantly, this approach enables the identification of high-attention segments within each sample sequence, facilitating subsequent biologically relevant analysis of specific functional amino acid regions.

### Multiple feature attribution comparison

To investigate the potential differences among various interpretability methods and gain a more comprehensive understanding of the mechanisms underlying transformer adapters in binary PPIs prediction, we employed both attention-based and feature attribution techniques to evaluate feature importance [30]. Attention-based methods provide insights into the specific features the model prioritizes during prediction, while feature attribution methods quantify the individual contributions of each feature to the final prediction outcome. By systematically analyzing feature importance using these complementary approaches and comparing their results, we aim to more effectively identify the key features driving model decisions, thereby ensuring that the predictions are not only accurate but also biologically meaningful [25, 54].

**Figure 8:**
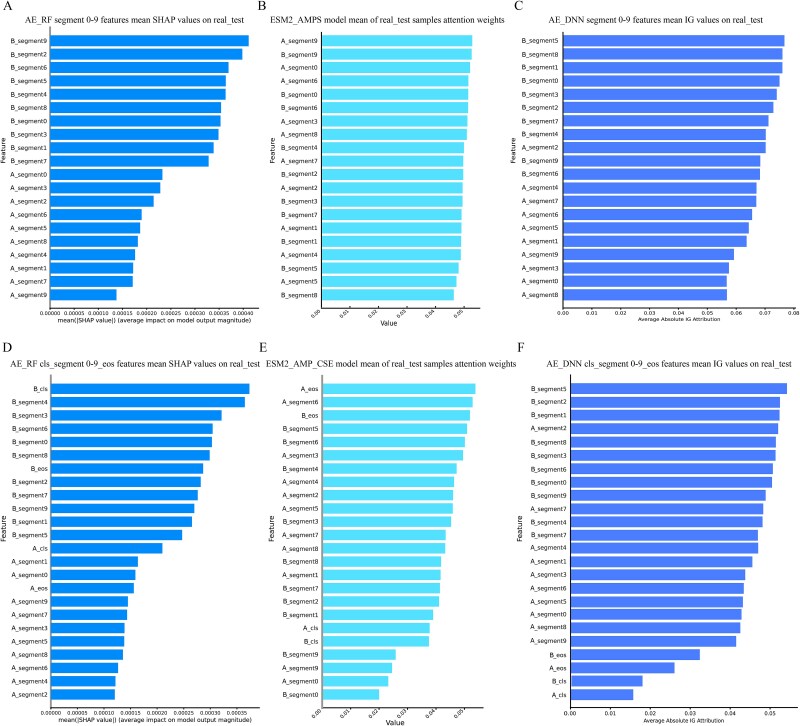
Comparison of interpretability approaches for feature importance on the real_test dataset. (A) Summary plot of tree SHAP values for segment features (A_segment0–9 and B_segment0–9) in the AE_RF model. (B) Average attention weights for segment features in the ESM2_AMPS model. (C) Summary plot of integrated gradients values for segment features (A_segment0–9 and B_segment0–9) in the AE_DNN model. (D) Summary plot of tree SHAP values for special token features (A_cls, A_eos, B_cls, B_eos) and segment features in the AE_RF model. (E) Average attention weights for special token features (A_cls, A_eos, B_cls, B_eos) and segment features in the ESM2_AMP_CSE model. (F) Summary plot of integrated gradients values for special token features (A_cls, A_eos, B_cls, B_eos) and segment features in the AE_DNN model.

Specifically, this research employs Tree SHAP and Integrated Gradient (IG) for feature attribution analysis, evaluating the contribution of segment features (A_segment0–9, B_segment0–9) and special tokens features (A_cls, B_cls, A_eos, B_eos) to PPIs predictions. On the real_test dataset, Tree SHAP applied in the AE_RF model (Fig. 8A, 8D) reveals that Protein B’s features generally have higher average SHAP values than Protein A’s. IG interpretation (applied in AE_DNN) also revealed a significant disparity in feature attribution scores between the two proteins, the features of Protein B consistently demonstrated substantially higher IG value compared to Protein A (Fig. 8C, 8F). Additionally, the Gini importance for the AE_RF model and SHAP feature importance for the AE_DNN model were computed. These results exhibited patterns consistent with those from Tree SHAP and IG, with Feature B consistently demonstrating higher importance values than Feature A (Fig. S13). The interpretability of the ESM2_AMP framework was validated using Integrated Gradients for feature importance calculation. Feature importance values were compared with attention weight quantifications, followed by Spearman correlation analyses between both importance measures for the corresponding 20 features (ESM2_AMPS) and 24 features (ESM2_AMP_CSE). Results demonstrated positive correlation between the two features of importance values in ESM2_AMPS, while ESM2_AMP_CSE showed negative correlation. This observation aligns with previous findings suggesting that special token features may disrupt the model’s attention to segment features. In contrast, an attention-based interpretability analysis of the ESM2_AMPS model shows minimal differences in feature importance between Protein A and B (Fig. 8B). For the attention results of the ESM2_AMP_CSE model, only A_segment0, A_segment9, B_segment0, and B_segment9 exhibit notably lower importance (Fig. 8E). These differences suggest that the self-attention mechanism captures richer information about protein interactions, potentially correlating with complex interaction patterns [55]. This is consistent with the nature of biological interaction signals, which are often indirect and implicit, requiring the model to infer subtle and context-dependent relationships. The observed variation in attention weights highlights the model’s ability to prioritize more informative segments, potentially reflecting the underlying biological mechanisms driving protein–protein interactions. This aligns with the hypothesis that attention mechanisms are particularly suited for modeling intricate and non-linear interaction patterns in biological systems.

### Correlation between feature attribution value and functional amino acid regions

Attention mechanisms compute pairwise importance scores between tokens. In ESM2_AMP, the different sections positional of segment tokens enables attention weights to reflect the contribution of different sequence sections positions to PPIs prediction [30]. Given that biological functional regions (such as domains [56]) are often associated with PPIs, analyzing high-attention features can reveal differences in functional intervals and their potential roles in PPIs [57]. In the ESM2_AMPS model, attention weights for segment features in the real_test dataset were analyzed to establish correlations with biologically functional regions. Samples were classified into four categories based on PPIs prediction: TP (True Positive), FP (False Positive), TN (True Negative), and FN (False Negative). For each class, the top three and bottom three segments with the highest and lowest average attention weights were selected, and their coverage of functional amino acid regions (*Domain* [43], *Region* [47], *Compositional bias* [44], *Repeat* [45], *Motif* [46]) was calculated (Table 5-6 and Table S1-S6).

**Table 5:** Functional amino acid sequence regions coverage in top three attention weight segments (TP class).

	*Domain* (%)	*Region* (%)	*Compositional bias* (%)	*Repeat* (%)	*Motif* (%)	All (%)
Top1	32.18	26.85	5.09	4.54	1.05	57.82
Top2	21.80	31.27	8.31	4.42	0.95	53.72
Top3	17.91	32.43	8.56	4.67	0.72	52.79
Average	23.96	30.18	7.32	4.54	0.91	54.78

**Table 6:** Functional amino acid sequence regions coverage in low three attention weight segments (TP class).

	*Domain* (%)	*Region* (%)	*Compositional bias* (%)	*Repeat* (%)	*Motif* (%)	All (%)
Low1	20.66	22.52	4.30	6.20	0.37	46.51
Low2	21.13	20.89	3.62	5.81	0.25	44.80
Low3	21.90	22.71	3.94	6.22	0.53	48.24
Average	21.23	22.04	3.95	6.08	0.38	46.52

In the TP class (Table 5 and Table 6), the top segment (Top 1) exhibited a 57.82% coverage of functional regions, which was 11.31% higher than the least segment (Low 1). *Domain* and *Region* showed higher coverage compared to other functional types, with *Domain* demonstrating a stronger correlation with attention weights than *Region* (Table 5). This suggests that the model effectively captures important functional regions relevant to prediction. The coverage of functional regions was higher in the TP class than in the FN (Table S1 and Table S2) and TN (Table S3 and Table S4) classes, indicating that the proportion of functional regions within segments plays a critical role in accurate prediction. In the FP class (Table S5 and Table S6), the coverage was comparable to that of the TP class, as the model improperly focused on segments with high functional region coverage, making these features important for learning negative samples. This emphasizes the model’s ability to distinguish between relevant and irrelevant functional regions across different sample types. Notably, some proteins (such as ‘P49336’, with a full sequence length of 464 amino acids and a domain interval of [21–335AA]) with long functional regions spanned multiple segments, resulting in moderate coverage even for segments with low attention weights.

**Figure 9:**
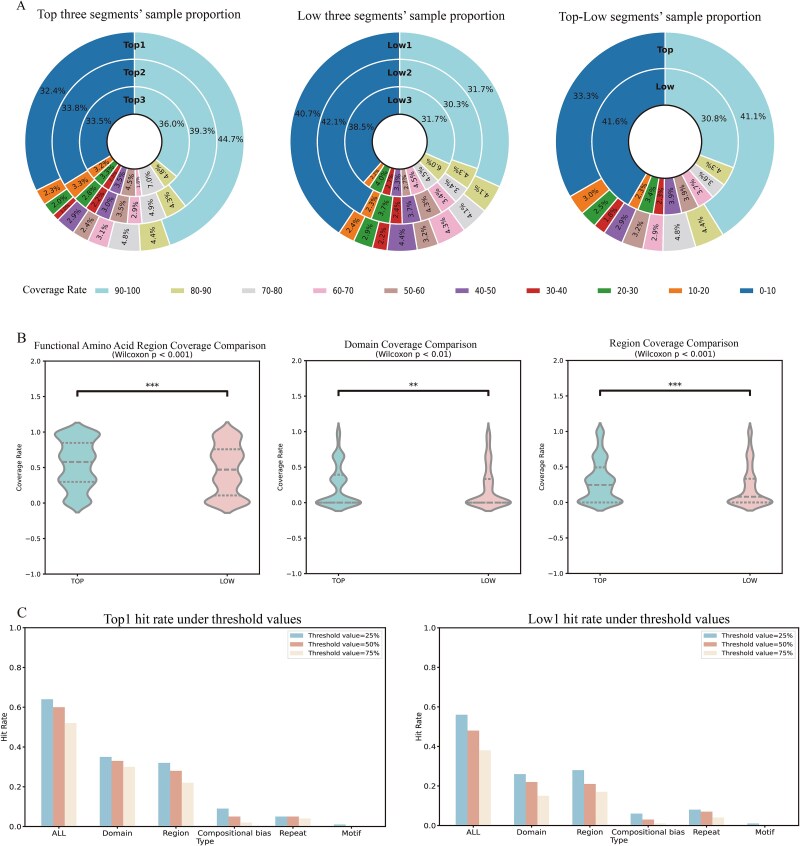
Analysis of functional amino acid region coverage and hit rates in the TP class. (A) Proportion of samples in different coverage intervals for top three, low three, and their average segments in the TP class. Colors denote coverage rate intervals, with values indicating the ratio of samples in each interval to the total real_test dataset sample size. (B) Wilcoxon rank-sum test (p < 0.01 or p < 0.001) for top and low segments of total functional amino acid region and two types (domain and region) in TP class. (C) Hit rates of functional amino acid regions for Top1 and Low1 high-attention-weight segments at 25%, 50%, and 75% thresholds in the TP class.

Based on the coverage analysis of segments in the real_test dataset, coverage levels were divided into ten intervals (0–10%, 10–20%, …, 90–100%) for further quantitative experiment. The specific value for each interval represents the proportion of samples within that coverage range relative to the total number of true positive (TP) class samples. For instance, in the TP class, the Top1 segment with high attention weights contained a greater number of samples with 90%–100% functional amino acid region coverage compared to Top2, Top3, and the low-attention segments (Fig. 9A, Tables S7-S8). For intervals of 30%–100%, the sample quantity decreased progressively from Top1 to Top3 segments. Furthermore, the top three segments exhibited higher sample quantities in the 20%–100% intervals compared to the low three (Fig. 9A, Table S9), further verifying the model’s capability to extract biological information from segment features. These findings align with the established significance of amino acid regions in protein interactions and function, reinforcing their relevance in PPIs prediction. Subsequently, for the coverage intervals of both *Domain* and *Region* types, the results (Fig. S14) demonstrate that *Domain* achieves a higher Top1 value in the 90–100% coverage interval compared to other intervals, and it also surpasses the Top1 value of *Region* in the same interval.

PPIs rely on critical residues or regions essential for protein function and interaction. Domains, disordered regions, motifs, compositional biases, and repeats all influence PPIs. Domains, such as the SH3 domain, represent functionally significant regions and serve as key modules for PPIs, playing essential roles in biological functions [43]. Disordered regions, which are highly flexible, are also crucial for PPIs, as they often mediate interactions, such as the p53-MRE11 interaction [47]. These functional amino acid regions can interact with multiple proteins, regulating various biological processes. In contrast, repeats, compositional biases, and motifs generally have less impact on PPIs due to their shorter lengths and limited functional relevance. Repeats are often conserved but do not always contribute significantly to function [58]. Compositional biases exhibit amino acid preferences but tend to play a minor role in functional regions. Motifs, while useful in specific interactions, are often scattered and may not fully align with functional regional standards, further limiting their contribution.

To validate the correlation between feature importance (quantified by attention weights) and functional amino acid region coverage, a rank-based statistical analysis compared coverage rates between the top three (Top1–Top3) and bottom three (Low1–Low3) feature importance segments in true positive (TP) samples. The Wilcoxon rank-sum test across functional amino acid region coverage, *Domain* coverage, *Region* coverage. The three results demonstrated statistically significant distributional differences (p < 0.01) between TOP and LOW (Fig. 9B). These robust findings not only confirm the strong association between feature importance and biologically functional regions but also demonstrate that the model effectively prioritizes sequence within functional domains.

Additionally, three coverage thresholds were established to compute the hit rates of functional amino acid regions across the four prediction categories: TP (Table 7-8 and Table S10-S13), TN (Table S14-S19), FP (Table S20-S25), and FN (Table S26-S31). A hit (1) was recorded if the coverage exceeded the threshold; otherwise, it was marked as a miss (0). For the TP class, the hit rates of the three segments with the highest attention weights (Top1, Top2, Top3) and the three segments with the lowest attention weights (Low1, Low2, Low3) were calculated, respectively. The results (Fig. S15) show that, under different thresholds, the hit rate of the Top1 segment is consistently the highest among all segments. Moreover, as the threshold increases, the gap in hit rates between the Top1 segment and the other segments widens. When the threshold was set to 50%, the total hit rate for the top three high-attention segments across the five functional types was 82.87%, which is 11.81% higher than that of the three lowest-attention segments (Table 7 and 8). The hit rate for *Domain* in the Top 1 segment was 32.82%, surpassing all other segments. As the threshold increased, the disparity in hit rates between *Domain* and *Region* became more pronounced (Fig. 9C, Table S10-S13), underscoring the critical role of Domain in the model. High-attention segments frequently corresponded to longer domain sequences, indicating that the model effectively captures this association. Notably, these domains (such as SH3 domain, Kinase domain) are known to participate in protein–protein interactions, suggesting that the model’s predictions are rooted in interaction mechanisms. Although *the Region*’s hit rate was comparable to *Domain*’s, it exhibited a weaker correlation with segment attention weights. Further validation was conducted using the InterPro database [59]. As *Region* annotations were not available, coverage analysis focused exclusively on total functional amino acid regions and *Domain*. The *Domain* analysis (Fig. S17) revealed that segments with the highest feature importance (corresponding to highest attention weights’ values) showed significantly greater domain coverage. This finding is consistent with the UniProt results.

**Table 7:** The types hit rate of attention weights values high top three segments in TP class (50% threshold).

	*Domain* (%)	*Region* (%)	*Compositional bias* (%)	*Repeat* (%)	*Motif* (%)	All (%)
Top1	32.82	27.86	4.60	4.78	0.27	59.96
Top2	22.72	31.38	6.67	4.69	0.45	55.18
Top3	18.39	32.91	6.85	4.78	0.18	54.28
Average	46.08	58.79	15.60	10.73	0.90	82.87

**Table 8:** The types hit rate of attention weights values high low three segments in TP class (50% threshold).

	*Domain* (%)	*Region* (%)	*Compositional bias* (%)	*Repeat* (%)	*Motif* (%)	All (%)
Low1	22.18	21.19	3.43	6.94	0.00	48.15
Low2	21.37	19.75	2.80	6.40	0.00	46.35
Low3	23.08	22.00	2.98	6.31	0.09	49.86
Average	38.68	38.41	6.85	12.17	0.09	71.06

The attention-based interpretability analysis, combined with functional region correlation, demonstrates that the ESM2_AMPS model effectively identifies *Domain* and *Region* relevant to PPIs. Constructed on the ESM2_AMP architecture and utilizing only segment features, the model aligns high-importance features with known functional domains, validating its ability to leverage local sequence information for reliable predictions. These insights not only confirm the model’s design but also deepen the understanding of protein interaction mechanisms, supporting applications in drug discovery and functional genomics.

## Discussion

In this research, we introduce ESM2_AMP, an interpretable deep learning framework for binary PPIs prediction. The ESM2_AMP framework is constructed based on segment features and special token embedding features, with the purpose of exploring the hidden mechanism embedding in ESM2. Its stand a computational reduced methods for deploy attention mechanism in fine tuning model for specifical downstream task, and unique to non-fine tuning in domain specific data interpretation methods [57]. Moreover, based on different feature integration methods (segment features or infusion of special tokens and segment features) constructed two models (ESM2_AMPS and ESM2_AMP_CSE) for PPIs prediction and the interpretation of model prediction decision mechanism. Overall, the ESM2_AMPS model achieved performance over ESM2_AMP_CSE in independent test set (real_test dataset) and five-fold cross-validation on the Multi_species_dataset and Ko_human_dataset. By conducting validation, on the more stringent human dataset (Bernett dataset), our approach demonstrated higher recall and F1 scores compared to previous PPIs models. Notably, our model achieves performance close to the current state-of-the-art (SOTA) TUnA model on the Bernett dataset. The overall performance significantly surpassed methods that did not utilize PLMs. Compared to residue-level feature representations [60], the segmental approach for token level feature representation displayed in this work substantially reduced computational resource consumption during finetune phase.

Through incorporating attention weight map as feature importance quantification method, ESM2_AMP accurately identifies key sequence segments that significantly impact prediction results and precisely correlate the model’s decision-making process with known protein domains. Moreover, multiple feature attribution methods (including SHAP and IG) were employed to provide multi-level and multi-dimensional model interpretation. The Tree SHAP feature-importance analysis revealed that the AE_RF model relies more heavily on the intrinsic features of the second protein (protein B). Consistently, Integrated Gradients (IG) analysis demonstrated that the AE_DNN model exhibits a similar preference toward protein B’s features.

These findings suggest that simply concatenating protein features, without incorporating interactive fusion mechanisms, is insufficient for capturing the global contextual information necessary for accurate PPIs prediction. This underscores the importance of integrating Transformer-based architectures into the ESM2_AMP framework, which enables modeling of complex, context-dependent interactions between protein sequences. Our proposed framework not only achieves good performance in binary PPIs prediction but also maintains high interpretability. By examining the overlap between high-attention sequence segments and annotated functional regions, we demonstrate that the model effectively identifies biologically relevant sequence patterns. Specifically, sequences enriched with functional domains are shown to play a decisive role in determining protein–protein interactions. This discovery advances interpretability methodologies for sequence-based PPIs prediction. Together, these findings offer novel insights for the optimization of computational algorithms and furnish experimental biologists with valuable reference for functional validation, highlighting the cross-disciplinary significance of our work.

There are also limitations in this research. Firstly, ESM2_AMP depends on the ESM2 model to extract features from amino acid sequences, which introduces some inference latency. Secondly, while fine-tuning the ESM2 protein language model as a feature extractor by dividing full-length protein sequences into ten equal segments is a straightforward approach, this method overlooks potential variations in segment ranges across sequences, which could impact the model’s ability to capture nuanced segment features and lacks the capacity to capture the diverse biological characteristics inherent within different proteins. In future research, we aim to refine the dynamic segmentation capability of large models to capture more precise biological information and explore model distillation and quantization techniques to optimize the model, thereby enhancing performance under resource-constrained conditions. Additionally, our predictive framework relies solely on primary sequence inputs, explicitly excluding protein structural information. This simplification enables efficient large-scale screening while acknowledging inherent limitations in modeling structural determinants. Future directions will incorporate graph-based neural architectures and predicted structural features (e.g., solvent accessibility, contact maps) to capture biophysical constraints. Finally, the interpretability methods based on attention mechanisms are different with feature attribution, triggering a challenge to comprehensively understand the model’s interpretability. For this direction, we will construct a unified interpretability framework to further enhance the robustness of model’s interpretability.

## Conclusion

In this study, we developed the ESM2_AMP deep learning framework for binary protein–protein interactions (PPIs) prediction. This framework leverages the ESM2 protein language model to extract special tokens features and segment features from protein sequences. Two PPI models, ESM2_AMPS and ESM2_AMP_CSE, were constructed based on different feature fusion strategies. The ESM2_AMP framework achieves adaptive fusion of cls, eos and segment features, demonstrating good performance in PPIs prediction. Notably, the ESM2_AMPS model, which relies solely on segment features, also performs exceptionally well and provides unique insights into PPIs mechanisms. Analysis using ESM2_AMPS on an independent test set revealed that the model’s decision-making aligns with known protein domains. Overall, our research confirms that the transformer-based adaptive model, combined with segmental feature fusion, effectively captures key information for binary PPIs prediction, enhancing model performance and offering new avenues for PPIs discovery using advanced deep learning frameworks.

**Key Points**

- An interpretable deep learning framework has been developed for predicting binary PPIs.
- The framework innovatively incorporates the importance of segment features, enabling interpretable analysis of PPIs prediction mechanisms and revealing new prediction model insights.
- A correlation has been identified between the predictive contribution of segment features and functional amino acid sequences.

### Glossary and list of abbreviations

PPIs, Protein–Protein Interactions; PLMs, Protein Language Models; MLP, Multilayer Perceptron; DNN, Deep Neural Network; SOTA, State-of-the-art; SHAP, Shapley Additive Explanations; MCC, Matthews Correlation Coefficient; AUC, Area Under the Curve; BERT, Bidirectional Encoder Representations from Transformers; AUROC, Area Under the Receiver Operating Characteristic; TP, True Positive; TN, True Negative; FN, False Negative; FP, False Positive; TPR, True Positive Rate; FPR, False Positive Rate; IG, Integrated Gradient; T5, Text-to-Text Transfer Transformer; GRU, Gated Recurrent Unit; AE, Autoencoder; RF, Random Forest; AE_RF, Autoencoder_ Random Forest

## Code and dataset available

Code and original datasets related to this article are available online at the following GitHub repository: https://github.com/ywwy-qn/ESM2_AMP. In addition, the raw data and corresponding protein language model (PLM) feature embeddings are available at https://figshare.com/articles/dataset/ESM2_AMP/28378157.

## Supplementary Material

supplementary_material_bbaf434

## References

[1] Greenblatt JF, Alberts BM, Krogan NJ. Discovery and significance of protein-protein interactions in health and disease. *Cell* 2024;187:6501–17. 10.1016/j.cell.2024.10.038 39547210 PMC11874950

[2] Tang T, Zhang X, Liu Y. et al. Machine learning on protein-protein interaction prediction: models, challenges and trends. *Brief Bioinform* 2023;24. 10.1093/bib/bbad076 36880207

[3] Hu L, Wang X, Huang YA. et al. A survey on computational models for predicting protein-protein interactions. *Brief Bioinform* 2021;22. 10.1093/bib/bbab036 33693513

[4] Kilisch M, Lytovchenko O, Schwappach B. et al. The role of protein–protein interactions in the intracellular traffic of the potassium channels TASK-1 and TASK-3. *Pflügers Archiv - European Journal of Physiology* 2015;467:1105–20. 10.1007/s00424-014-1672-2 25559843

[5] Dunham B, Ganapathiraju MK. Benchmark evaluation of protein-protein interaction prediction algorithms. *Molecules* 2021;27(1). 10.3390/molecules27010041 35011283 PMC8746451

[6] Fields S . Song O-k, a novel genetic system to detect protein–protein interactions. *Nature* 1989;340:245–6. 10.1038/340245a0 2547163

[7] Lin J-S, Lai E-M. Protein–protein interactions: Co-immunoprecipitation, in bacterial protein secretion systems: Methods and protocols. In: Journet L, Cascales E (eds.), *Springer*, pp. 211–9. New York: New York, NY, 2017. 10.1007/978-1-0716-3445-5_18

[8] Guo Y, Yu L, Wen Z. et al. Using support vector machine combined with auto covariance to predict protein-protein interactions from protein sequences. *Nucleic Acids Res* 2008;36:3025–30. 10.1093/nar/gkn159 18390576 PMC2396404

[9] Shen J, Zhang J, Luo X. et al. Predicting protein-protein interactions based only on sequences information. *Proc Natl Acad Sci U S A* 2007;104:4337–41. 10.1073/pnas.0607879104 17360525 PMC1838603

[10] Hashemifar S, Neyshabur B, Khan AA. et al. Predicting protein-protein interactions through sequence-based deep learning. *Bioinformatics* 2018;34:i802–10. 10.1093/bioinformatics/bty573 30423091 PMC6129267

[11] Chen M, Ju CJ, Zhou G. et al. Multifaceted protein-protein interaction prediction based on Siamese residual RCNN. *Bioinformatics* 2019;35:i305–14. 10.1093/bioinformatics/btz328 31510705 PMC6681469

[12] Yao Y, Du X, Diao Y. et al. An integration of deep learning with feature embedding for protein-protein interaction prediction. *PeerJ* 2019;7:e7126. 10.7717/peerj.7126 31245182 PMC6585896

[13] Elnaggar A, Heinzinger M, Dallago C. et al. ProtTrans: toward understanding the language of life through self-supervised learning. *IEEE Trans Pattern Anal Mach Intell* 2022;44:7112–27. 10.1109/TPAMI.2021.3095381 34232869

[14] Dang TH, Vu TA. xCAPT5: protein-protein interaction prediction using deep and wide multi-kernel pooling convolutional neural networks with protein language model. *BMC Bioinformatics* 2024;25:106. 10.1186/s12859-024-05725-6 38461247 PMC10924985

[15] Ko YS, Parkinson J, Liu C. et al. TUnA: an uncertainty-aware transformer model for sequence-based protein-protein interaction prediction. *Brief Bioinform* 2024;25. 10.1093/bib/bbae359 PMC11269822 39051117

[16] Gao Z, Jiang C, Zhang J. et al. Hierarchical graph learning for protein–protein interaction. *Nat Commun* 2023;14:1093. 10.1038/s41467-023-36736-1 36841846 PMC9968329

[17] Vaswani A, Shazeer N, Parmar N. et al. Attention is all you need. arXiv e-prints. 2017. arXiv:1706.03762. 10.48550/arXiv.1706.03762

[18] Devlin J, Chang M-W, Lee K. et al. BERT: pre-training of deep bidirectional transformers for language understanding. arXiv e-prints. 2018. arXiv:1810.04805. 10.48550/arXiv.1810.04805

[19] Lin Z, Akin H, Rao R. et al. Evolutionary-scale prediction of atomic-level protein structure with a language model. *Science* 2023;379:1123–30. 10.1126/science.ade2574 36927031

[20] Beltran A, Jiang XE, Shen Y. et al. Site-saturation mutagenesis of 500 human protein domains. *Nature* 2025;637:885–94. 10.1038/s41586-024-08370-4 39779847 PMC11754108

[21] Mastropietro A, Pasculli G, Bajorath J. Learning characteristics of graph neural networks predicting protein–ligand affinities. *Nature Machine Intelligence* 2023;5:1427–36. 10.1038/s42256-023-00756-9

[22] Chan KY, Abu-Salih B, Qaddoura R. et al. Deep neural networks in the cloud: review, applications, challenges and research directions. *Neurocomputing* 2023;545:126327. 10.1016/j.neucom.2023.126327

[23] Pan X-Y, Zhang Y-N, Shen H-B. Large-scale prediction of human protein−protein interactions from amino acid sequence based on latent topic features. *J Proteome Res* 2010;9:4992–5001. 10.1021/pr100618t 20698572

[24] Bernett J, Blumenthal DB, List M. Cracking the black box of deep sequence-based protein-protein interaction prediction. *Brief Bioinform* 2024;25. 10.1093/bib/bbae076 PMC10939362 38446741

[25] Chen V, Yang M, Cui W. et al. Applying interpretable machine learning in computational biology—pitfalls, recommendations and opportunities for new developments. *Nat Methods* 2024;21:1454–61. 10.1038/s41592-024-02359-7 39122941 PMC11348280

[26] Lundberg SM, Lee S-I. A Unified Approach to Interpreting Model Predictions, in Proceedings of the 31st International Conference on Neural Information Processing Systems. Long Beach, California, USA: Curran Associates Inc, 2017. 4768–77. https://dl.acm.org/doi/10.5555/3295222.3295230

[27] Zhao N, Zhuo M, Tian K. et al. Protein-protein interaction and non-interaction predictions using gene sequence natural vector. *Commun Biol* 2022;5:652. 10.1038/s42003-022-03617-0 35780196 PMC9250521

[28] Blohm P, Frishman G, Smialowski P. et al. Negatome 2.0: a database of non-interacting proteins derived by literature mining, manual annotation and protein structure analysis. *Nucleic Acids Res* 2014;42:D396–400. 10.1093/nar/gkt1079 24214996 PMC3965096

[29] Sanders P, Schulz C. KaHIP v3.00 -- Karlsruhe High Quality Partitioning -- User Guide arXiv e-prints 2013. arXiv:1311.1714. 10.48550/arXiv.1311.1714

[30] Luo Z, Wang R, Sun Y. et al. Interpretable feature extraction and dimensionality reduction in ESM2 for protein localization prediction. *Brief Bioinform* 2024;25. 10.1093/bib/bbad534 PMC10818170 38279650

[31] Subasi A . Chapter 3 - machine learning techniques. In: Subasi A (ed.), *Practical Machine Learning for Data Analysis Using Python*, pp. 91–202. Academic Press, 2020. 10.1016/B978-0-12-821379-7.00003-5

[32] Zhang YJ, Luo Z, Sun Y. et al. From beasts to bytes: revolutionizing zoological research with artificial intelligence. *Zool Res* 2023;44:1115–31. 10.24272/j.issn.2095-8137.2023.263 37933101 PMC10802096

[33] Loshchilov I, Hutter F. Fixing weight decay regularization in Adam. ArXiv. 2017. abs/1711.05101. 10.48550/arXiv.1711.05101

[34] Akiba T, Sano S, Yanase T. et al. Optuna: A Next-Generation Hyperparameter Optimization Framework arXiv e-prints 2019. arXiv:1907.10902. 10.48550/arXiv.1907.10902

[35] Zhao C, Li X. Linearization of ReLU Activation Function for Neural Network-Embedded Optimization:Optimal Day-Ahead Energy Scheduling arXiv e-prints 2023. arXiv:2310.01758. 10.48550/arXiv.2310.01758

[36] He K, Zhang X, Ren S. et al. Delving Deep into Rectifiers: Surpassing Human-Level Performance on ImageNet Classification arXiv e-prints 2015. arXiv:1502.01852. 10.48550/arXiv.1502.01852

[37] Chicco D, Jurman G. The Matthews correlation coefficient (MCC) should replace the ROC AUC as the standard metric for assessing binary classification. *BioData Min* 2023;16:4. 10.1186/s13040-023-00322-4 36800973 PMC9938573

[38] Hinton GE, Zemel RS. Autoencoders, Minimum Description Length and Helmholtz Free Energy, in Proceedings of the 7th International Conference on Neural Information Processing Systems. Denver, Colorado: Morgan Kaufmann Publishers Inc, 1993. 3–10. https://dl.acm.org/doi/10.5555/2987189.2987190

[39] Lundberg SM, Erion G, Chen H. et al. From local explanations to global understanding with explainable AI for trees. *Nat Mach Intell* 2020;2:56–67. 10.1038/s42256-019-0138-9 32607472 PMC7326367

[40] Bank D, Koenigstein N, Giryes R. Autoencoders arXiv e-prints 2020. arXiv:2003.05991. 10.48550/arXiv.2003.05991

[41] Rigatti SJ . Random Forest. *J Insur Med* 2017;47:31–9. 10.17849/insm-47-01-31-39.1 28836909

[42] Nicodemus KK . Letter to the editor: on the stability and ranking of predictors from random forest variable importance measures. *Brief Bioinform* 2011;12:369–73. 10.1093/bib/bbr016 21498552 PMC3137934

[43] Wang D, Liu G, Meng Y. et al. The configuration of GRB2 in protein interaction and signal transduction. *Biomolecules* 2024;14. 10.3390/biom14030259 PMC10968029 38540680

[44] Mier P, Paladin L, Tamana S. et al. Disentangling the complexity of low complexity proteins. *Brief Bioinform* 2020;21:458–72. 10.1093/bib/bbz007 30698641 PMC7299295

[45] Chakrabarty B, Parekh N. Sequence and structure-based analyses of human Ankyrin repeats. *Molecules* 2022;27. 10.3390/molecules27020423 35056738 PMC8781854

[46] Ivarsson Y, Jemth P. Affinity and specificity of motif-based protein-protein interactions. *Curr Opin Struct Biol* 2019;54:26–33. 10.1016/j.sbi.2018.09.009 30368054

[47] Usluer S, Galhuber M, Khanna Y. et al. Disordered regions mediate the interaction of p53 and MRE11. *Biochim Biophys Acta Mol Cell Res* 2024;1871:119654. 10.1016/j.bbamcr.2023.119654 38123020

[48] UniProt C . UniProt: the universal protein knowledgebase in 2023. *Nucleic Acids Res* 2023;51:D523–31. 10.1093/nar/gkac1052 36408920 PMC9825514

[49] Chung J, Gulcehre C, Cho K. et al. Empirical Evaluation of Gated Recurrent Neural Networks on Sequence Modeling arXiv e-prints 2014. arXiv:1412.3555. 10.48550/arXiv.1412.3555

[50] Sledzieski S, Singh R, Cowen L. et al. D-SCRIPT translates genome to phenome with sequence-based, structure-aware, genome-scale predictions of protein-protein interactions. *Cell Syst* 2021;12:969–982 e6. 10.1016/j.cels.2021.08.010 34536380 PMC8586911

[51] Singh R, Devkota K, Sledzieski S. et al. Topsy-Turvy: integrating a global view into sequence-based PPI prediction. *Bioinformatics* 2022;38:i264–72. 10.1093/bioinformatics/btac258 35758793 PMC9235477

[52] Raganato A, Tiedemann J. An Analysis of Encoder Representations in Transformer-Based Machine Translation. in BlackboxNLP@EMNLP 2018. 10.18653/v1/W18-5431

[53] Zhang S, Fan R, Liu Y. et al. Applications of transformer-based language models in bioinformatics: a survey. *Bioinform Adv* 2023;3:vbad001. 10.1093/bioadv/vbad001 36845200 PMC9950855

[54] Dickinson Q, Meyer JG. Positional SHAP (PoSHAP) for interpretation of machine learning models trained from biological sequences. *PLoS Comput Biol* 2022;18:e1009736. 10.1371/journal.pcbi.1009736 35089914 PMC8797255

[55] Dehimi NEH, Tolba Z. Attention mechanisms in deep learning : Towards explainable artificial intelligence. In: in 2024 6th International Conference on Pattern Analysis and Intelligent Systems (PAIS), 2024. 10.1109/PAIS62114.2024.10541203

[56] Meynard-Piganeau B, Fabbri C, Weigt M. et al. Generating interacting protein sequences using domain-to-domain translation. *Bioinformatics* 2023;39. 10.1093/bioinformatics/btad401 PMC10329493 37399105

[57] Nayar G, Tartici A, Altman RB. Paying Attention to Attention: High Attention Sites as Indicators of Protein Family and Function in Language Models bioRxiv 2024. 2024.12.13.628435. 10.1101/2024.12.13.628435 PMC12448987 40938959

[58] Andrade MA, Perez-Iratxeta C, Ponting CP. Protein repeats: structures, functions, and evolution. *J Struct Biol* 2001;134:117–31. 10.1006/jsbi.2001.4392 11551174

[59] Blum M, Andreeva A, Florentino LC. et al. InterPro: the protein sequence classification resource in 2025. *Nucleic Acids Res* 2025;53:D444–56. 10.1093/nar/gkae1082 39565202 PMC11701551

[60] Liu D, Young F, Lamb KD. et al. PLM-interact: extending protein language models to predict protein-protein interactions. *bioRxiv* 2024. 2024.11.05.622169. 10.1101/2024.11.05.622169 PMC12559430 41145424

