# Supplementary material for: ESM2_AMP: an interpretable framework for protein–protein interactions prediction and biological mechanism discovery
Source: Brief Bioinform. 2025 Aug 28;26(4):bbaf434. doi: 10.1093/bib/bbaf434 (PMC12392411; doi:10.1093/bib/bbaf434)
Supplement: supplementary_material_bbaf434 [file supplementary_material_bbaf434.docx]

**SUPPLEMENTARY MATERIAL**

**Supplementary Methods**

**Hyperparameter tuning setting**

In ESM2_AMPS model training process, the learning rate was tuned within the range of 1e-5 to 1e-3, while the weight decay was adjusted between 1e-4 and 1e-2. For the MLP module, the first hidden layer size was varied from 480 to 640 with a step size of 160, and the second hidden layer size was explored from 80 to 320 with a step size of 80. ESM2_AMP_CSE model maintained these parameters but extended the weight decay range to 1e-4-1e-1. In ESM2_DPM model training process, the learning rate was tuned within the range of 1e-6 to 1e-5, while the weight decay was adjusted between 1e-3 and 1e-1. For the DNN module, the first hidden layer size varied from 960 to 1280 with a step size of 320, the second hidden layer size was explored from 320 to 640 with a step size of 160, and the last layer was 40 to 160 with 60 steps.

Models hyperparameter results:

**ESM2_AMPS**: learning rate: 0.00012188013399710655; weight decay: 0.00029515893139888446; hidden1_dim: 640; hidden2_dim: 240.

**ESM2_AMP_CSE**: learning rate: 9.588973797963086e-05; weight decay: 0.01914594615905292; hidden1_dim: 480; hidden2_dim: 240.

**ESM2_DPM**: learning rate: 9.876545286721473e-06; weight decay: 0.015248379909099413; hidden1_dim: 1280; hidden2_dim: 480; hidden3_dim: 100.

**Supplementary Results**

Training and inference time consumption

The training and inference time of ESM2_AMPS and ESM2_GRU models were evaluated on the Bernett dataset, with training conducted on 2 NVIDIA V100 GPUs in parallel (35 epochs total) and inference performed on an NVIDIA A100 GPU. Results indicate ESM2_AMPS required 9,535.059 seconds for training and 65.31 seconds for inference, while ESM2_GRU completed training in 8,305.269 seconds with 62.26 seconds inference time. Compared to the TUnA baseline (13,329 seconds training, 438 seconds inference), both ESM2 models showed substantially faster performance, with ESM2_GRU being the most efficient. All timings may vary slightly across hardware configurations.

For feature extraction using ESM2, processing times were measured with different length constraints on protein sequences. When limiting sequences to 512 amino acids (13,300 proteins processed on an NVIDIA A100), the computation time was 3,243.32 seconds. For sequences between 512-1,024 amino acids (5,600 proteins), the inference time was 1,613.47 seconds.

**Supplementary Figure**

**
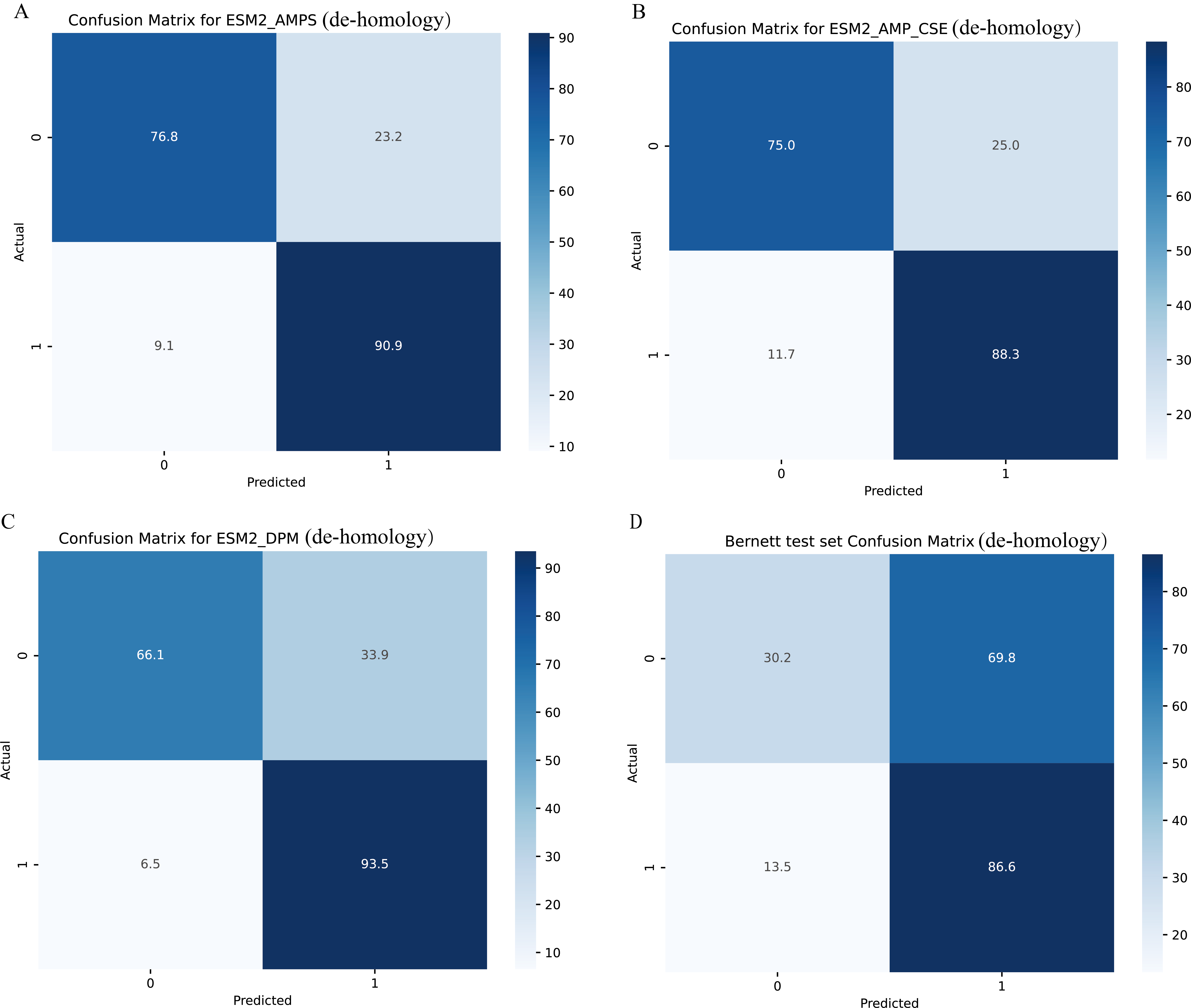
**

**Figure S1.** Confusion matrices for the de-homology test sets. (A) Confusion matrix of the real_test dataset (de-homology) on the ESM2_AMPS model; (B) Confusion matrix of the real_test dataset (de-homology) on the ESM2_AMP_CSE model; (C) Confusion matrix of the real_test dataset (de-homology) on the ESM2_DPM model; (D) Confusion matrix of the Bernett test set (de-homology) on the ESM2_AMPS model.

**
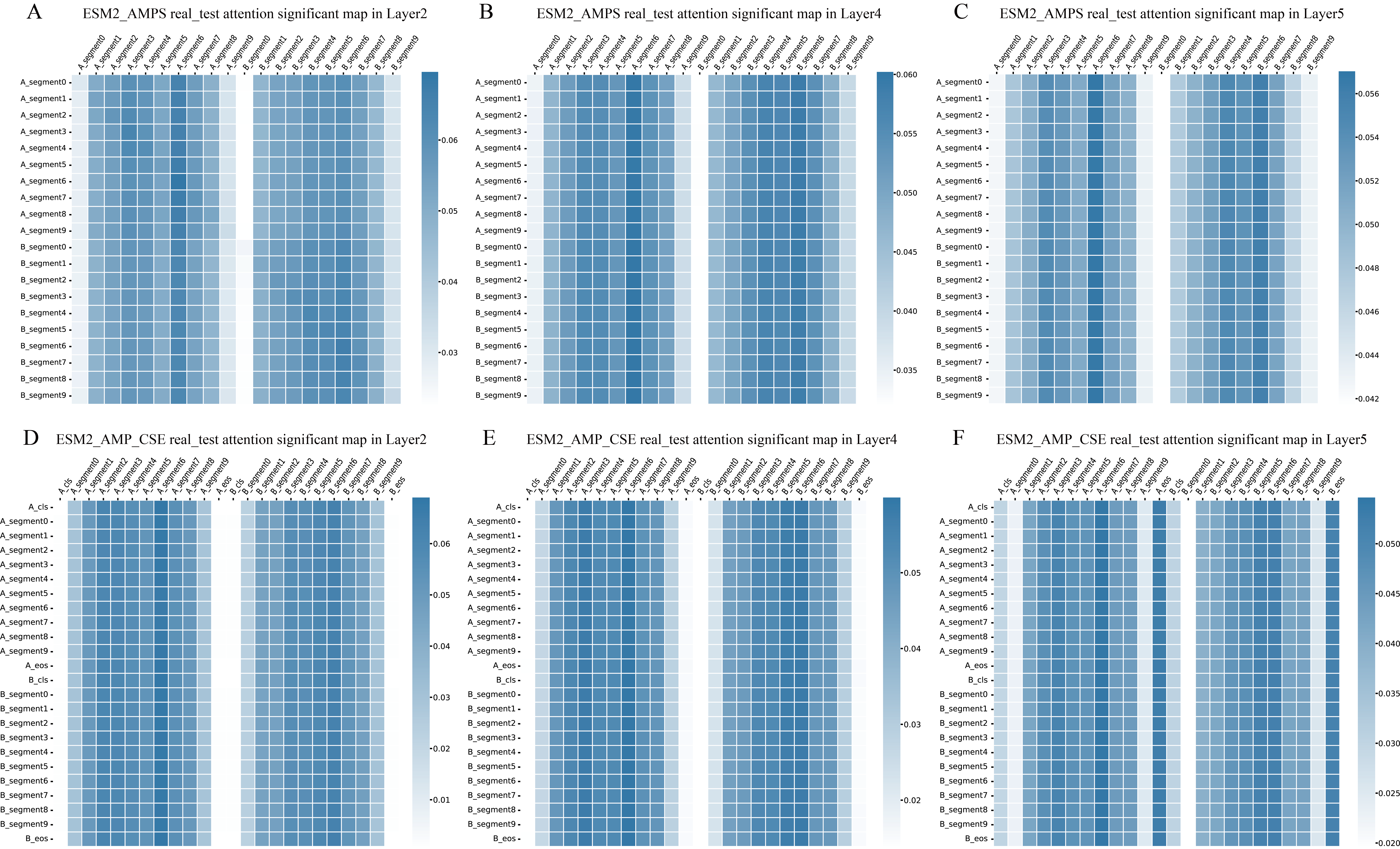
**

**Figure S2** The average attention significant maps of protein pairs on real_test dataset. (A-C) Attention significant maps of layers 2, 4, and 5 in the ESM2_AMPS model on real_test dataset. (D-F) Attention significant maps of layers 2, 4, and 5 in the ESM2_AMP_CSE model on real_test dataset.

**

**

**Figure S3** The average attention significant maps of layers 1-6 in the ESM2_AMPS model on Bernett dataset.

**
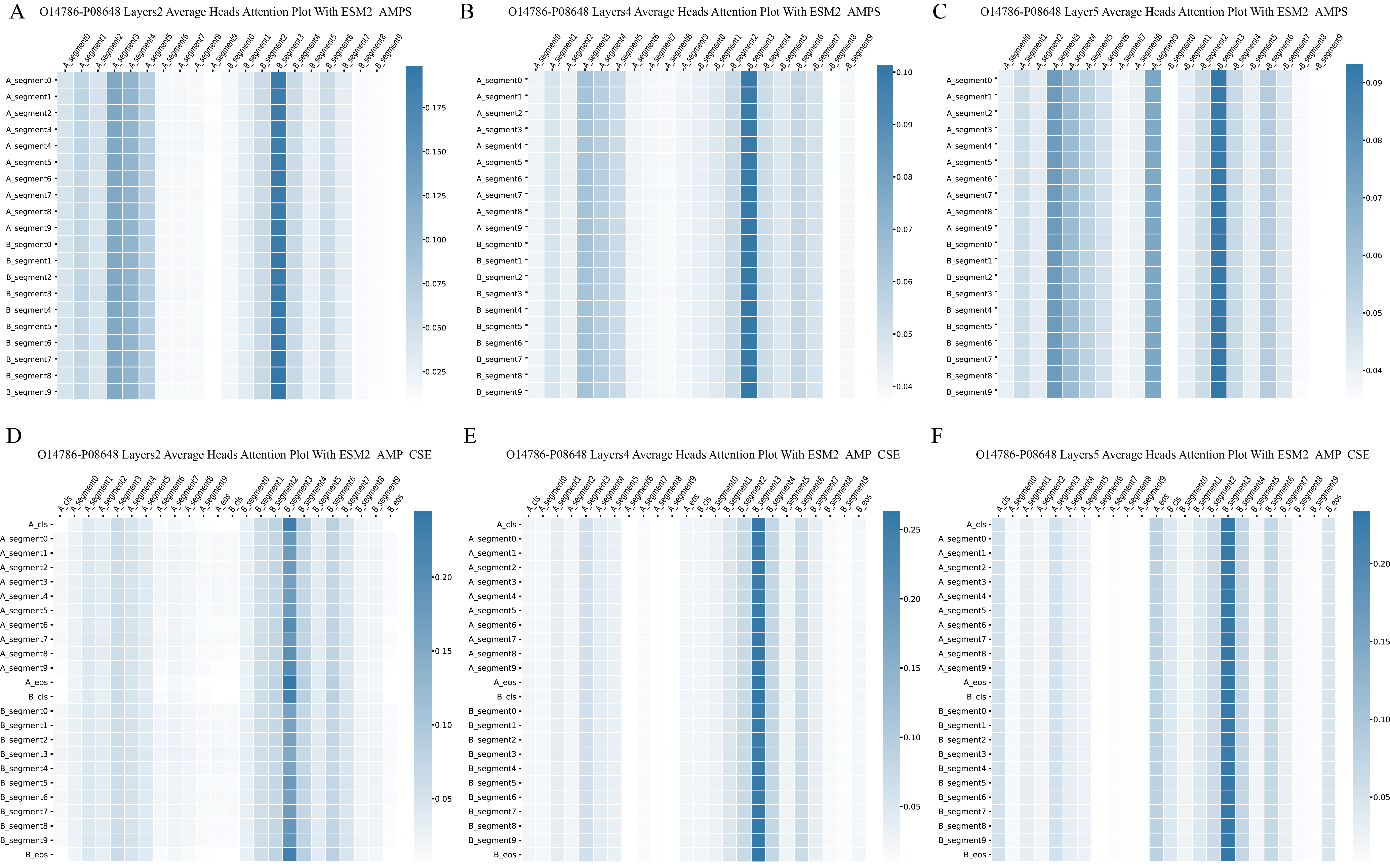
**

**Figure S4** The layer-wise attention significant maps of “O14786-P08648” protein pair in different models. (A-C) Layer-wise attention significance maps (layers 2/4/5) for protein pair “O14786-P08648” in ESM2_AMPS. (D-F) Layer-wise attention significance maps (layers 2/4/5) for protein pair “O14786-P08648” in ESM2_AMP_CSE.


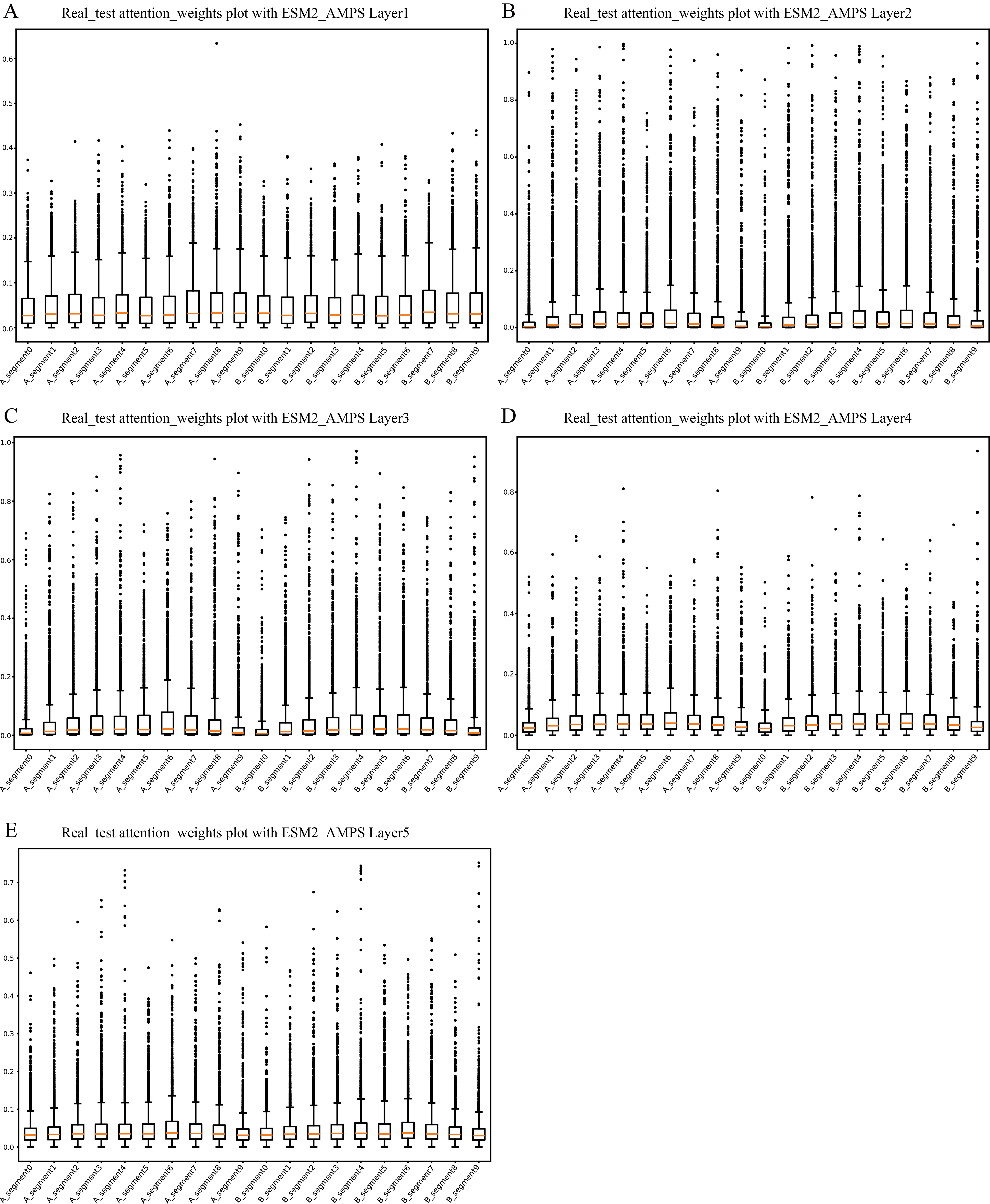


**Figure S5** Boxplot of average attention weights in the layers 1-5 of the ESM2_AMPS model on the real_test dataset.

**
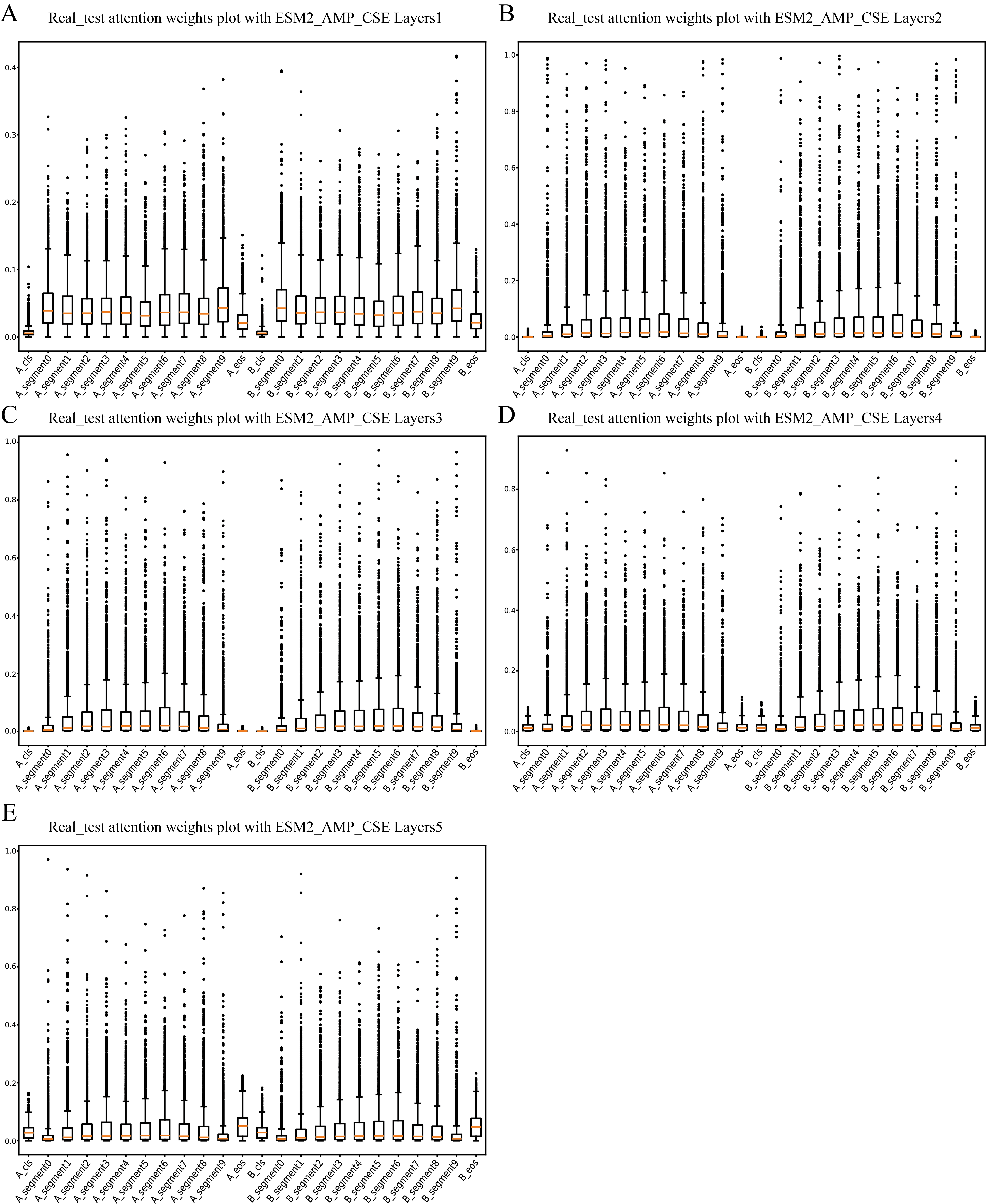
**

**Figure S6** Boxplot of average attention weights in the layers 1-5 of the ESM2_AMP_CSE model on the real_test dataset.

**
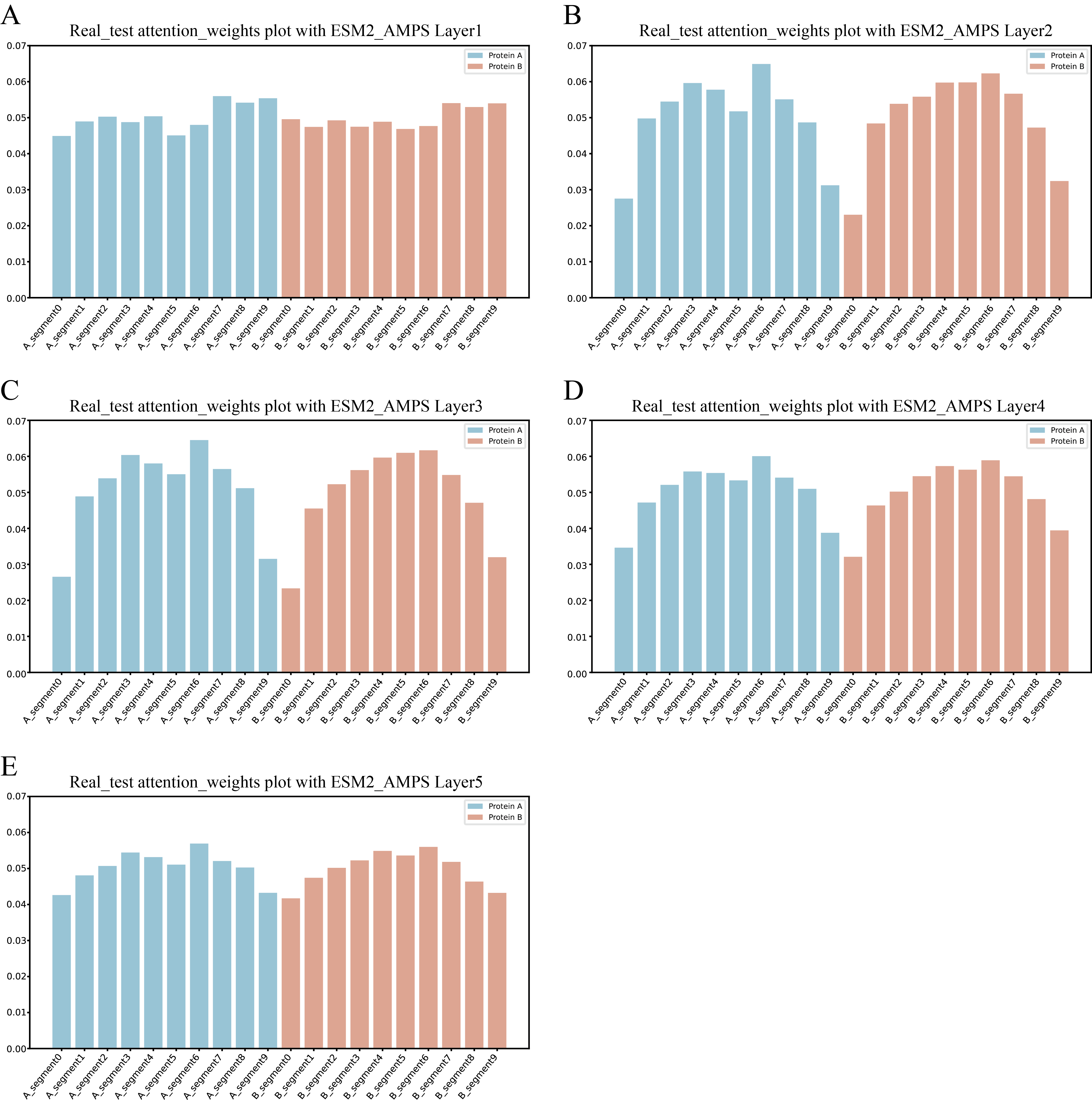
**

**Figure S7** Bar chart of mean value average attention weights in the layers 1-5 of the ESM2_AMPS model on the real_test dataset.


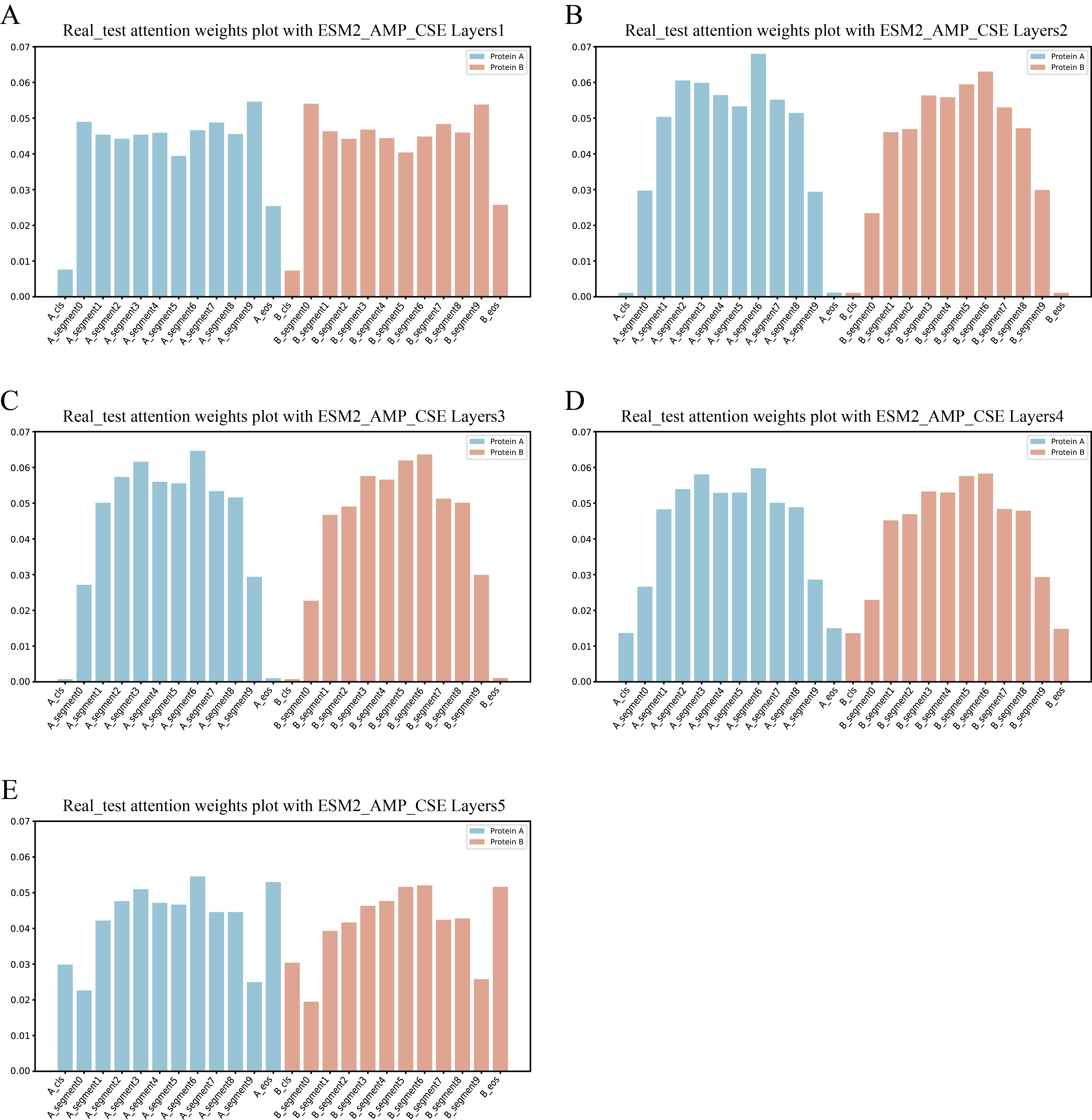


**Figure S8** Bar chart of mean value average attention weights in the layer 1-5 of the ESM2_AMP_CSE model on the real_test dataset.

**
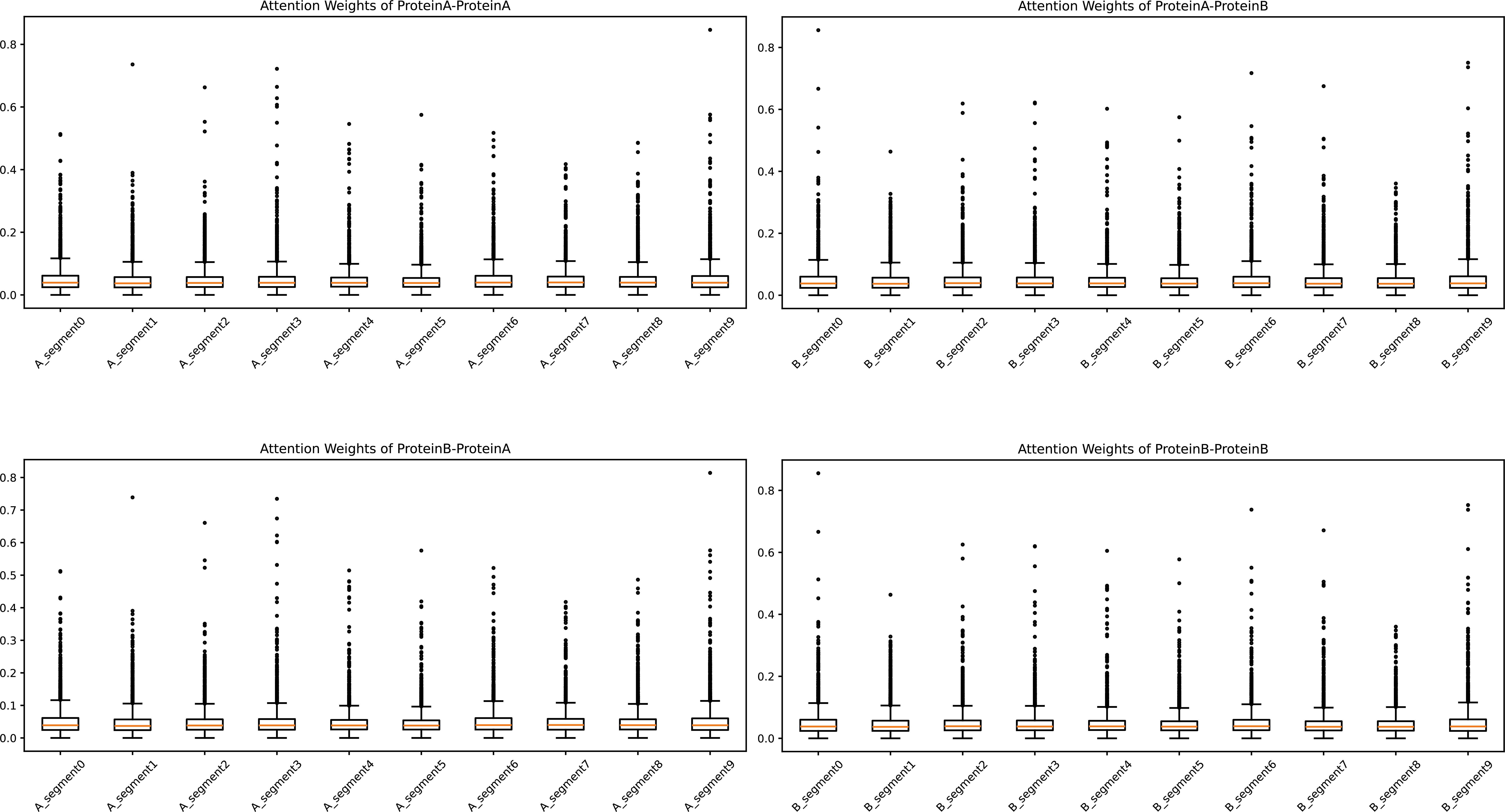
**

**Figure S9** Boxplot of different measures average attention weights in the sixth layer of the ESM2_AMPS model on the real_test dataset. (A) Weight values of Protein A features on Protein A features. (B) Weight values of Protein A features on Protein B features. (C) Weight values of Protein B features on Protein A features. (D) Weight values of Protein B features on Protein B features.

**
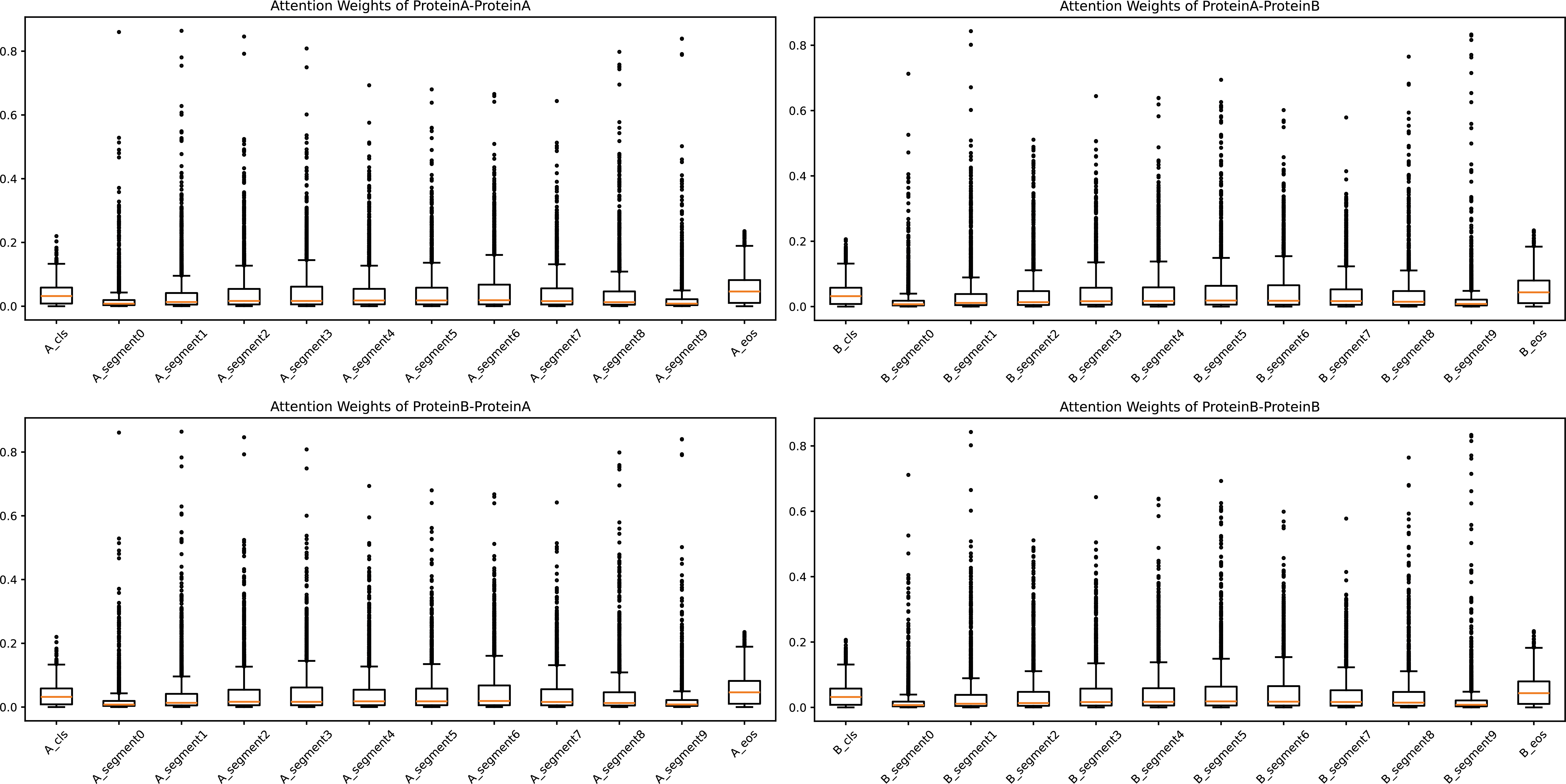
**

**Figure S10**Boxplot of different measures average attention weights in the sixth layer of the ESM2_AMP_CSE model on the real_test dataset. (A) Weight values of Protein A features on Protein A features. (B) Weight values of Protein A features on Protein B features. (C) Weight values of Protein B features on Protein A features. (D) Weight values of Protein B features on Protein B features.

**
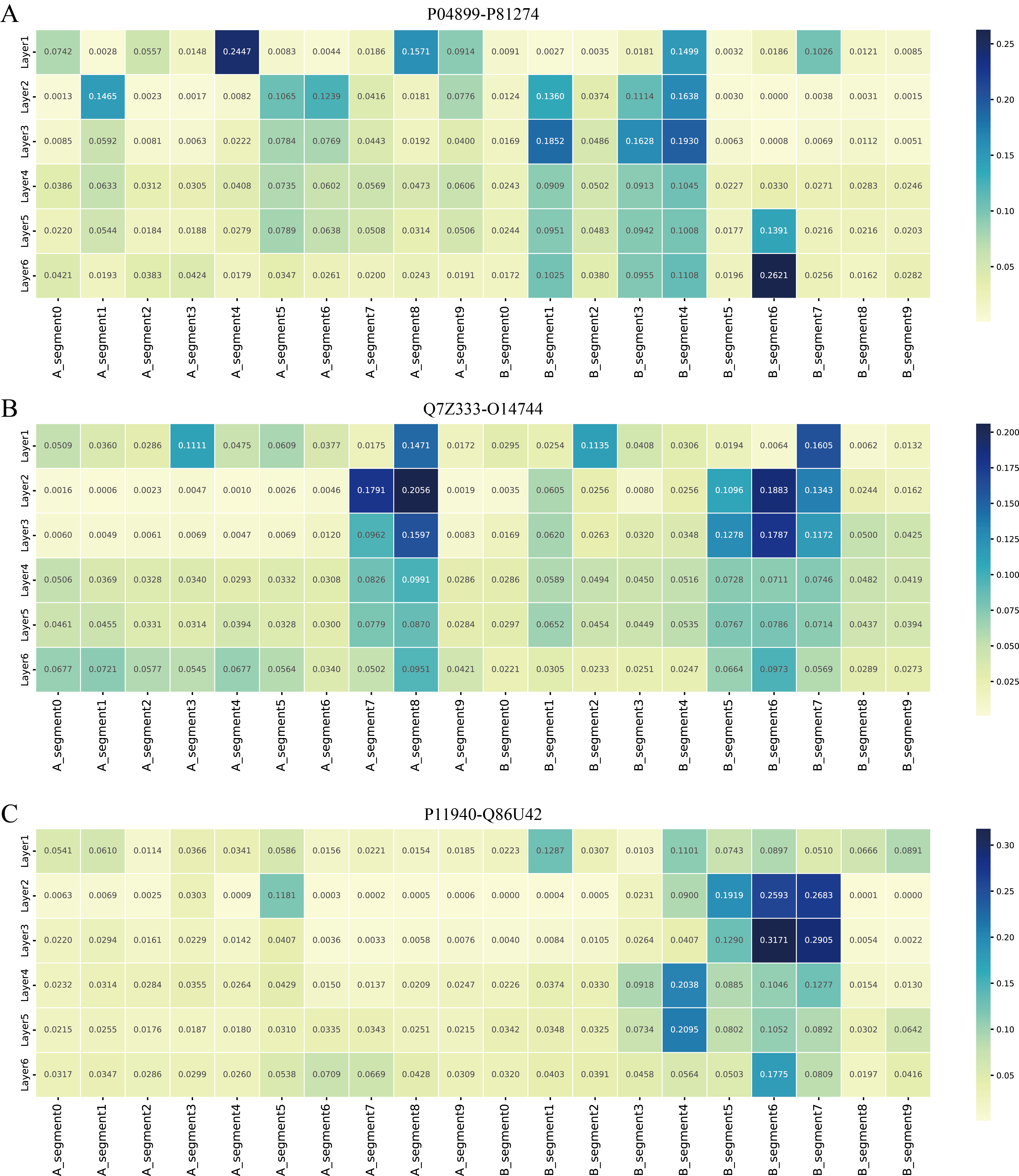
**

**Figure S11** Average weight values across six encoder layers in ESM2_AMPS for multiple samples. (A) Sample “P04899-P81274” across six encoder layers. (B) Sample “Q7Z333-O14744” across six encoder layers. (C) Sample “P11940-Q86U42” across six encoder layers.


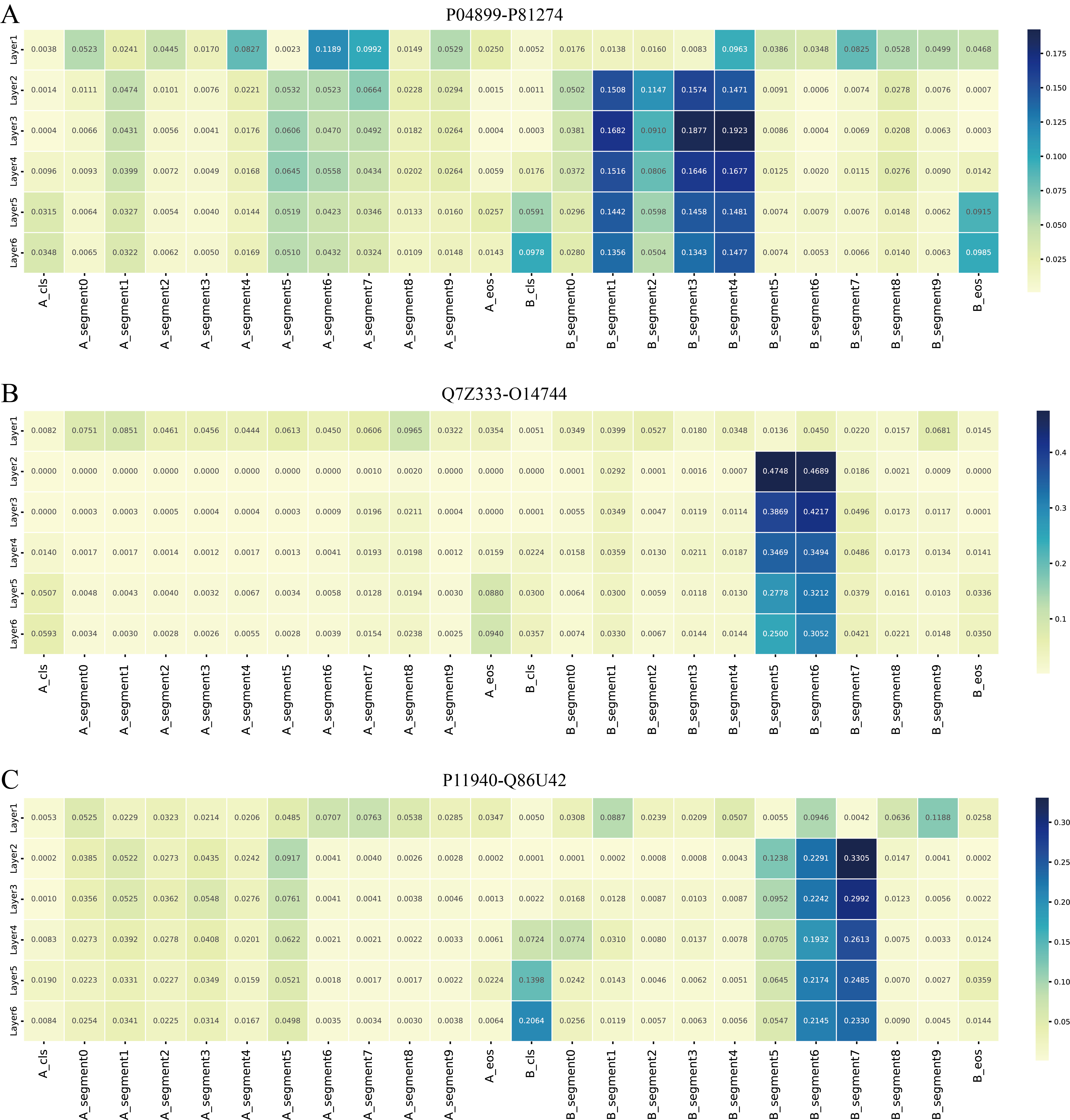


**Figure S12** Average weight values across six encoder layers in ESM2_AMP_CSE for multiple samples. (A) Sample “P04899-P81274” across six encoder layers. (B) Sample “Q7Z333-O14744” across six encoder layers. (C) Sample “P11940-Q86U42” across six encoder layers.


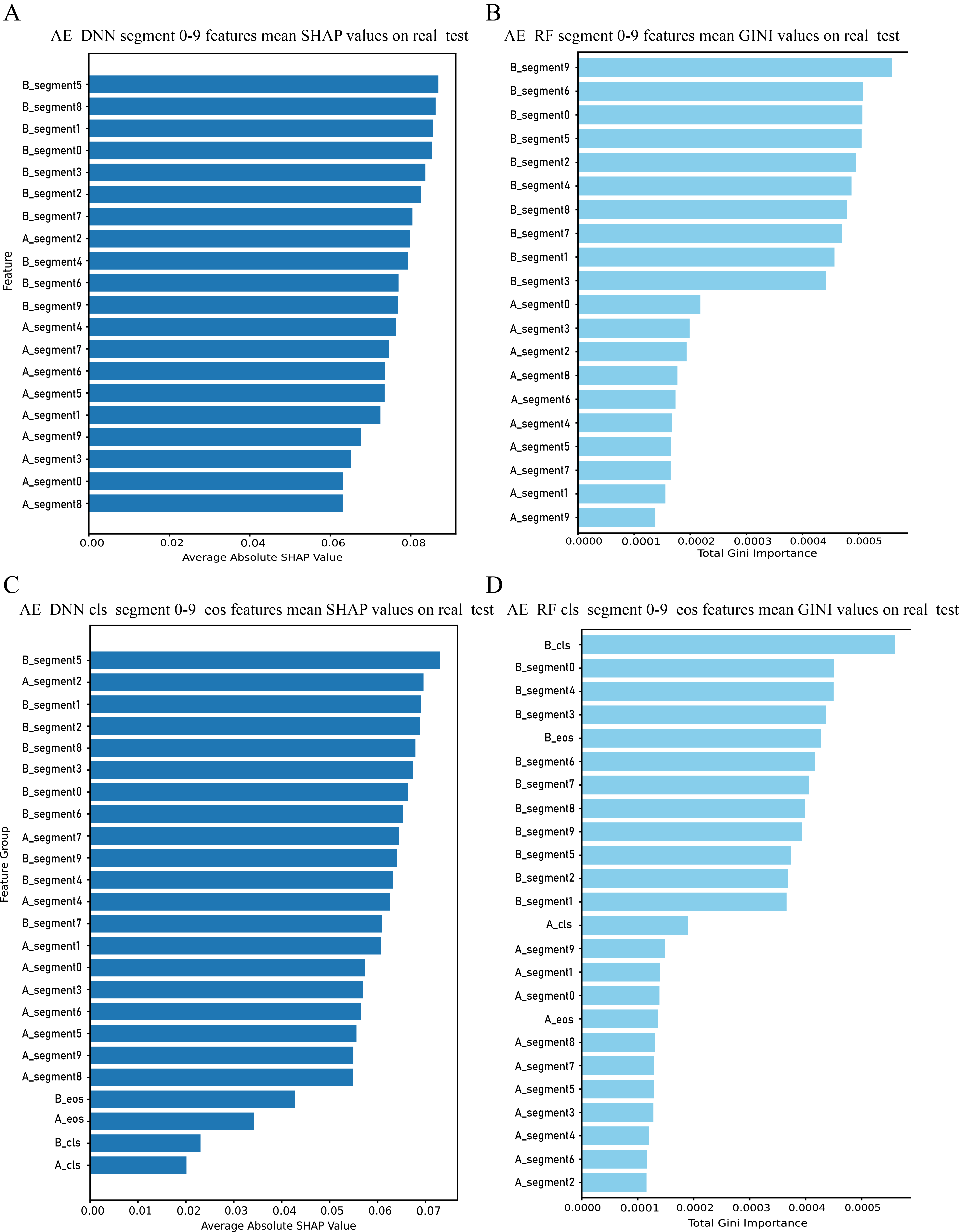


**Figure S13** Comparison of interpretability approaches for feature importance on the real_test dataset. (A) Summary plot of SHAP values for segment features (A_segment0-9 and B_segment0-9) in the AE_DNN model. (B) Summary plot of GINI inportant values for segment features (A_segment0-9 and B_segment0-9) in the AE_RF model. (C) Summary plot of SHAP values for special tokens represented features (A_cls, A_eos, B_cls, B_eos) and segment features in the AE_DNN model.

(D) Summary plot of GINI inportant values for special tokens represented features (A_cls, A_eos, B_cls, B_eos) and segment features in the AE_RF model.

**

**

**Figure S14.** Analysis the two types of functional amino acid region coverage and in the TP Class. (A) Proportion of samples in different *Domain* coverage intervals for top three, low three, and their average segments in the TP class. (B) Proportion of samples in different *Region* coverage intervals for top three, low three, and their average segments in the TP class.


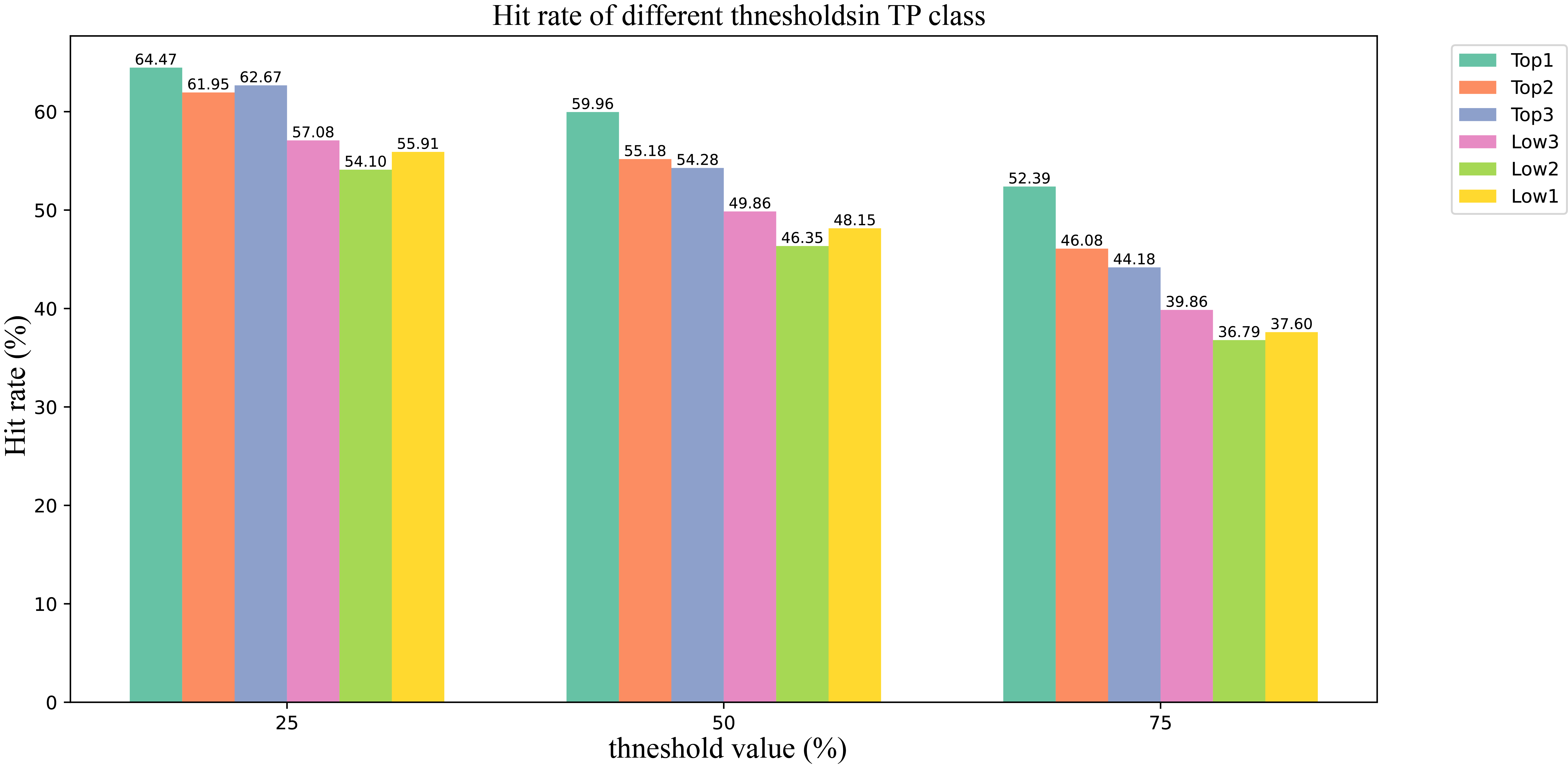


**Figure S15.** Functional amino acid region hit ratio of six segments (Top1, Top2, Top3, Low1, Low2, Low3) of true positive (TP) class under different thresholds.


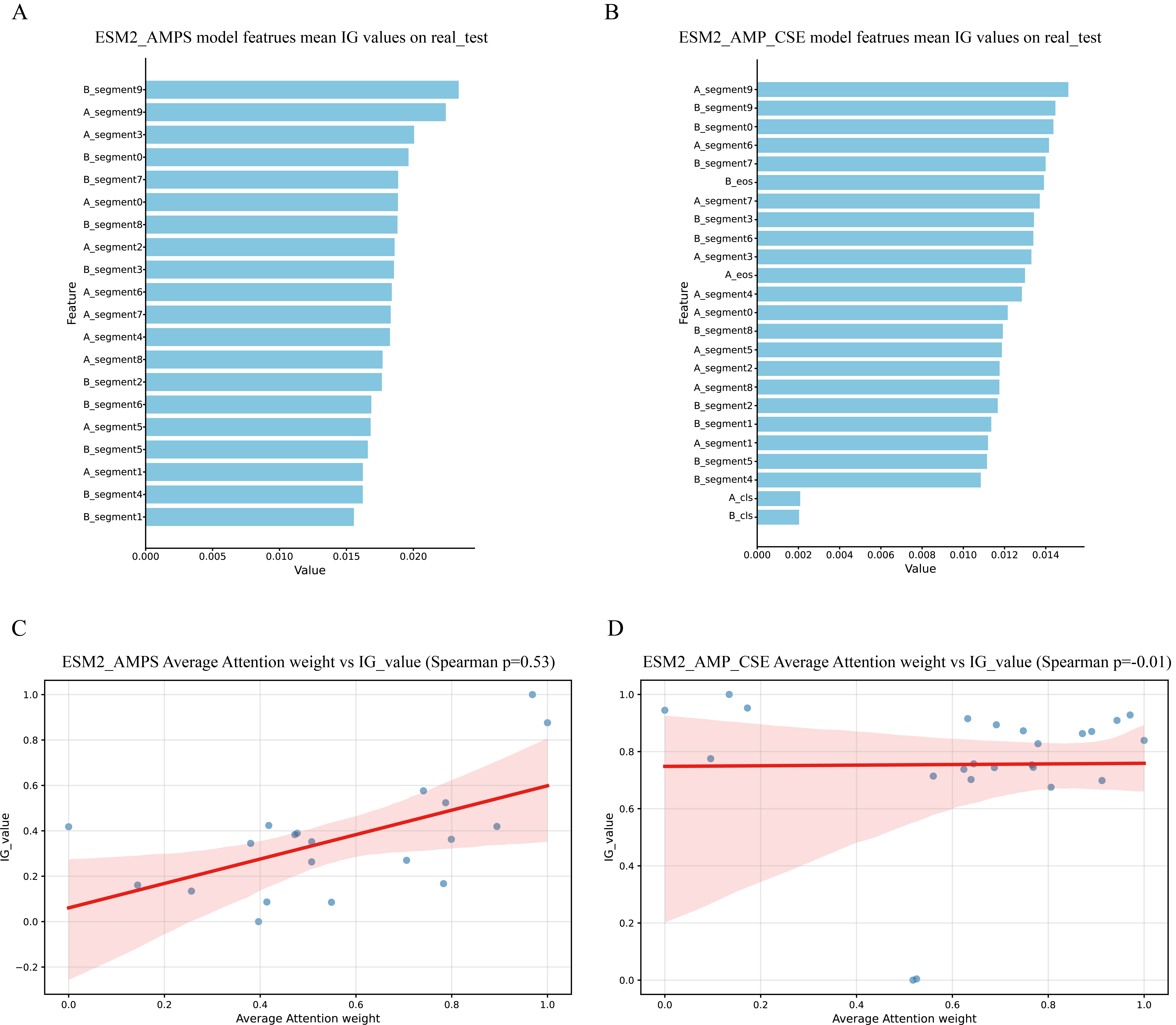


**Figure S16.** Comparison of interpretability approaches for feature importance on the real_test dataset with models and spearman correlation analysis. (A) Summary plot of IG values for segment features (A_segment0-9 and B_segment0-9) in the ESM2_AMPS model. (B) Summary plot of IG values for special token features (A_cls, A_eos, B_cls, B_eos) and segment features in the ESM2_AMP_CSE model. (C) Spearman correlate analysis of average attention weight and IG value in the ESM2_AMPS model.

(D) Spearman correlate analysis of average attention weight and IG value in the ESM2_AMP_CSE model.


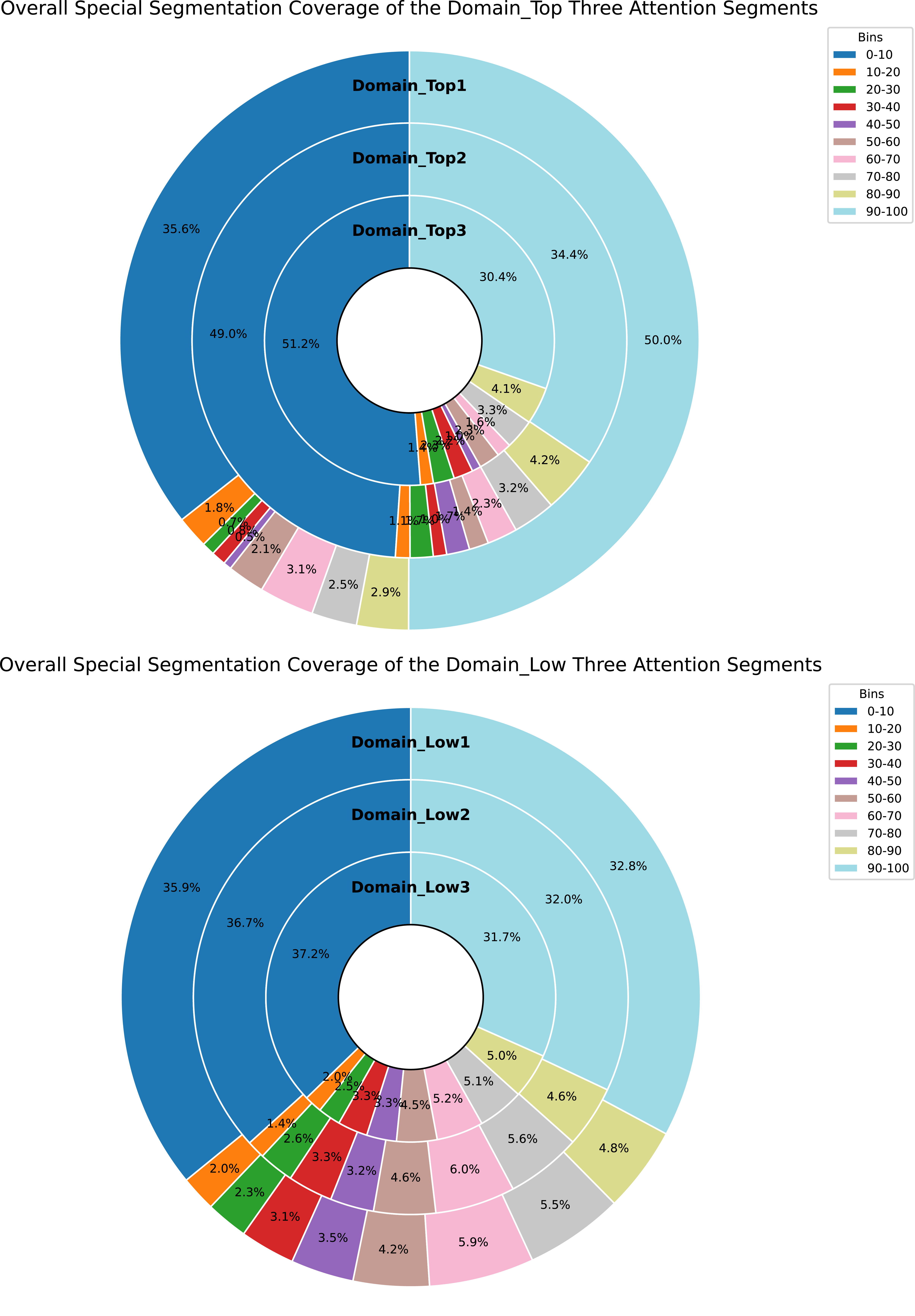


**Figure S17.** Analysis the different segments’ coverage of *Domain* in the TP Class from InterPro database.

**Table S1. Functional amino acid sequence regions coverage in top three attention weight segments (FN class)**

|  | ***Domain* (%)** | ***Region* (%)** | ***Compositional bias* (%)** | ***Repeat* (%)** | ***Motif* (%)** | **All (%)** |
| --- | --- | --- | --- | --- | --- | --- |
| Top1 | 19.25 | 24.93 | 5.60 | 0 | 0.17 | 43.11 |
| Top2 | 13.48 | 26.75 | 6.04 | 5.57 | 0.31 | 42.46 |
| Top3 | 20.29 | 19.95 | 4.10 | 9.97 | 0.16 | 45.32 |
| Average | 17.67 | 23.88 | 5.25 | 5.18 | 0.21 | 43.63 |

**Table S2. Functional amino acid sequence regions coverage in low three attention weight segments (FN class)**

|  | ***Domain* (%)** | ***Region* (%)** | ***Compositional bias* (%)** | ***Repeat* (%)** | ***Motif* (%)** | **All (%)** |
| --- | --- | --- | --- | --- | --- | --- |
| Low1 | 9.66 | 16.52 | 2.24 | 5.47 | 0.05 | 28.05 |
| Low2 | 13.05 | 20.41 | 1.17 | 6.07 | 0.05 | 34.94 |
| Low3 | 6.95 | 16.87 | 3.48 | 7.46 | 0.42 | 27.56 |
| Average | 9.89 | 17.93 | 2.30 | 6.33 | 0.17 | 30.18 |

**Table S3. Functional amino acid sequence regions coverage in top three attention weight segments (TN class)**

|  | ***Domain* (%)** | ***Region* (%)** | ***Compositional bias* (%)** | ***Repeat* (%)** | ***Motif* (%)** | **All (%)** |
| --- | --- | --- | --- | --- | --- | --- |
| Top1 | 28.47 | 31.21 | 6.77 | 2.71 | 0.84 | 55.36 |
| Top2 | 30.70 | 28.19 | 5.30 | 3.99 | 0.62 | 56.81 |
| Top3 | 30.27 | 27.28 | 4.87 | 4.37 | 0.65 | 56.34 |
| Average | 29.81 | 28.89 | 5.65 | 3.69 | 0.70 | 56.17 |

**Table S4. Functional amino acid sequence regions coverage in low three attention weight segments (TN class)**

|  | ***Domain* (%)** | ***Region* (%)** | ***Compositional bias* (%)** | ***Repeat* (%)** | ***Motif* (%)** | **All (%)** |
| --- | --- | --- | --- | --- | --- | --- |
| Low1 | 20.70 | 24.49 | 6.64 | 1.26 | 0.66 | 43.10 |
| Low2 | 23.33 | 25.06 | 6.24 | 2.39 | 0.47 | 46.71 |
| Low3 | 24.86 | 22.10 | 5.06 | 2.81 | 0.73 | 46.77 |
| Average | 22.96 | 23.88 | 5.98 | 2.15 | 0.62 | 45.53 |

**Table S5.** **Functional amino acid sequence regions coverage in top three attention weight segments (FP class)**

|  | ***Domain* (%)** | ***Region* (%)** | ***Compositional bias* (%)** | ***Repeat* (%)** | ***Motif* (%)** | **All (%)** |
| --- | --- | --- | --- | --- | --- | --- |
| Top1 | 35.36 | 24.74 | 4.18 | 4.86 | 0.53 | 58.94 |
| Top2 | 32.39 | 28.68 | 5.95 | 3.13 | 0.50 | 60.37 |
| Top3 | 24.83 | 31.28 | 8.29 | 3.67 | 0.84 | 56.84 |
| Average | 30.86 | 28.23 | 6.14 | 3.89 | 0.62 | 58.72 |

**Table S6. Functional amino acid sequence regions coverage in low three attention weight segments (FP class)**

|  | ***Domain* (%)** | ***Region* (%)** | ***Compositional bias* (%)** | ***Repeat* (%)** | ***Motif* (%)** | **All (%)** |
| --- | --- | --- | --- | --- | --- | --- |
| Low1 | 32.30 | 21.82 | 3.42 | 3.81 | 0.83 | 52.61 |
| Low2 | 33.78 | 21.11 | 4.18 | 3.36 | 0.87 | 53.53 |
| Low3 | 34.61 | 24.79 | 4.68 | 3.58 | 0.63 | 57.70 |
| Average | 33.56 | 22.57 | 4.09 | 3.58 | 0.78 | 54.61 |

**Table S7. Supplementary table showing the percentage distribution of samples in different intervals for Top three (Figure 9A)**

| **Coverage intervals** | **Top1 (%)** | **Top2 (%)** | **Top3 (%)** |
| --- | --- | --- | --- |
| 90-100 | 44.7 | 39.3 | 36 |
| 80-90 | 4.4 | 4.3 | 4.8 |
| 70-80 | 4.8 | 4.9 | 7 |
| 60-70 | 3.1 | 2.9 | 1.8 |
| 50-60 | 2.4 | 3.5 | 4.5 |
| 40-50 | 2.9 | 3 | 3.5 |
| 30-40 | 1 | 2.2 | 2.3 |
| 20-30 | 2 | 3.8 | 3.3 |
| 10-20 | 2.3 | 3.3 | 3.2 |
| 0-10 | 32.4 | 33.8 | 33.5 |

**Table S8. Supplementary table showing the percentage distribution of samples in different intervals for Low three (Figure 9A)**

| **Coverage intervals** | **Low1 (%)** | **Low2 (%)** | **Low3 (%)** |
| --- | --- | --- | --- |
| 90-100 | 31.7 | 30.3 | 31.7 |
| 80-90 | 4.1 | 4.3 | 6 |
| 70-80 | 4.1 | 3.4 | 4.5 |
| 60-70 | 4.3 | 3.4 | 4.5 |
| 50-60 | 3.2 | 4.3 | 2.7 |
| 40-50 | 4.4 | 3.7 | 3.3 |
| 30-40 | 2.2 | 2.4 | 2.7 |
| 20-30 | 2.9 | 3.7 | 4 |
| 10-20 | 2.4 | 2.3 | 2.2 |
| 0-10 | 40.7 | 42.1 | 38.5 |

**Table S9. Supplementary table showing the percentage distribution of samples in different Top-Low intervals for Figure 9A.**

| **Coverage intervals** | **Top (%)** | **Low (%)** |
| --- | --- | --- |
| 90-100 | 41.1 | 30.8 |
| 80-90 | 4.4 | 4.3 |
| 70-80 | 4.8 | 3.6 |
| 60-70 | 2.9 | 3.7 |
| 50-60 | 3.2 | 3.9 |
| 40-50 | 2.9 | 3.9 |
| 30-40 | 1.8 | 2.3 |
| 20-30 | 2.5 | 3.4 |
| 10-20 | 3 | 2.3 |
| 0-10 | 33.3 | 41.6 |

**Table S10. The types hit rate of Attention Weights values high top three segments in TP class (25% threshold)**

|  | ***Domain* (%)** | ***Region* (%)** | ***Compositional bias* (%)** | ***Repeat* (%)** | ***Motif* (%)** | **All (%)** |
| --- | --- | --- | --- | --- | --- | --- |
| Top1 | 34.90 | 32.01 | 9.11 | 5.14 | 0.99 | 64.47 |
| Top2 | 24.08 | 37.15 | 13.98 | 5.41 | 1.44 | 61.95 |
| Top3 | 19.93 | 40.04 | 13.62 | 6.04 | 0.81 | 62.67 |
| Average | 47.43 | 67.27 | 29.49 | 12.26 | 2.98 | 87.38 |

**Table S11. The types hit rate of Attention Weights values high top three segments in TP class (75% threshold)**

|  | ***Domain* (%)** | ***Region* (%)** | ***Compositional bias* (%)** | ***Repeat* (%)** | ***Motif* (%)** | **All (%)** |
| --- | --- | --- | --- | --- | --- | --- |
| Top1 | 30.03 | 22.18 | 1.71 | 4.41 | 0.00 | 52.39 |
| Top2 | 19.75 | 24.98 | 2.98 | 3.88 | 0.00 | 46.08 |
| Top3 | 15.78 | 24.62 | 2.52 | 4.33 | 0.00 | 44.18 |
| Average | 42.92 | 46.80 | 6.58 | 9.92 | 0.00 | 74.93 |

**Table S12. The types hit rate of Attention Weights values low three segments in TP class (25% threshold)**

|  | ***Domain* (%)** | ***Region* (%)** | ***Compositional bias* (%)** | ***Repeat* (%)** | ***Motif* (%)** | **All (%)** |
| --- | --- | --- | --- | --- | --- | --- |
| Low1 | 25.70 | 28.40 | 5.95 | 8.48 | 0.63 | 55.91 |
| Low2 | 26.96 | 26.15 | 5.68 | 8.03 | 0.27 | 54.10 |
| Low3 | 27.50 | 27.68 | 5.68 | 8.21 | 0.81 | 57.08 |
| Average | 43.55 | 48.15 | 12.62 | 14.43 | 1.62 | 75.83 |

**Table S13. The types hit rate of Attention Weights values low three segments in TP class (75% threshold)**

|  | ***Domain* (%)** | ***Region* (%)** | ***Compositional bias* (%)** | ***Repeat* (%)** | ***Motif* (%)** | **All (%)** |
| --- | --- | --- | --- | --- | --- | --- |
| Low1 | 14.79 | 16.50 | 1.26 | 4.42 | 0.00 | 37.60 |
| Low2 | 15.78 | 15.33 | 0.99 | 4.06 | 0.00 | 36.79 |
| Low3 | 16.14 | 16.95 | 1.53 | 4.87 | 0.00 | 39.86 |
| Average | 29.85 | 30.66 | 3.16 | 8.75 | 0.00 | 63.03 |

**Table S14. The types hit rate of Attention Weights values high top three segments in TN class (25% threshold)**

|  | ***Domain* (%)** | ***Region* (%)** | ***Compositional bias* (%)** | ***Repeat* (%)** | ***Motif* (%)** | **All (%)** |
| --- | --- | --- | --- | --- | --- | --- |
| Top1 | 32.94 | 38.34 | 12.38 | 3.43 | 1.05 | 66.01 |
| Top2 | 33.99 | 34.26 | 8.56 | 5.14 | 1.19 | 65.22 |
| Top3 | 34.39 | 32.28 | 8.96 | 5.27 | 1.45 | 64.56 |
| Average | 58.76 | 64.82 | 26.48 | 8.83 | 3.16 | 89.33 |

**Table S15. The types hit rate of Attention Weights values high top three segments in TN class (50% threshold)**

|  | ***Domain* (%)** | ***Region* (%)** | ***Compositional bias* (%)** | ***Repeat* (%)** | ***Motif* (%)** | **All (%)** |
| --- | --- | --- | --- | --- | --- | --- |
| Top1 | 28.46 | 32.54 | 6.06 | 2.90 | 0.26 | 56.52 |
| Top2 | 32.02 | 27.67 | 4.74 | 4.48 | 0.00 | 57.58 |
| Top3 | 31.09 | 27.93 | 3.69 | 5.01 | 0.13 | 58.10 |
| Average | 56.26 | 57.58 | 13.57 | 8.70 | 0.40 | 84.58 |

**Table S16. The types hit rate of Attention Weights values high top three segments in TN class (75% threshold)**

|  | ***Domain* (%)** | ***Region* (%)** | ***Compositional bias* (%)** | ***Repeat* (%)** | ***Motif* (%)** | **All (%)** |
| --- | --- | --- | --- | --- | --- | --- |
| Top1 | 24.90 | 25.03 | 1.19 | 2.24 | 0.13 | 46.25 |
| Top2 | 27.93 | 22.40 | 2.24 | 3.16 | 0.00 | 50.59 |
| Top3 | 25.69 | 22.53 | 1.45 | 3.82 | 0.13 | 48.75 |
| Average | 50.99 | 46.51 | 4.61 | 7.25 | 0.26 | 79.05 |

**Table S17. The types hit rate of Attention Weights values low three segments in TN class (25% threshold)**

|  | ***Domain* (%)** | ***Region* (%)** | ***Compositional bias* (%)** | ***Repeat* (%)** | ***Motif* (%)** | **All (%)** |
| --- | --- | --- | --- | --- | --- | --- |
| Low1 | 26.35 | 30.83 | 10.67 | 1.84 | 1.05 | 52.96 |
| Low2 | 28.85 | 31.62 | 9.09 | 3.43 | 1.05 | 57.31 |
| Low3 | 30.57 | 26.88 | 8.96 | 4.08 | 0.92 | 56.39 |
| Average | 48.22 | 52.96 | 23.19 | 5.80 | 3.03 | 80.24 |

**Table S18. The types hit rate of Attention Weights values low three segments in TN class (50% threshold)**

|  | ***Domain* (%)** | ***Region* (%)** | ***Compositional bias* (%)** | ***Repeat* (%)** | ***Motif* (%)** | **All (%)** |
| --- | --- | --- | --- | --- | --- | --- |
| Low1 | 21.34 | 23.45 | 5.93 | 1.58 | 0.00 | 42.82 |
| Low2 | 23.72 | 23.72 | 5.27 | 2.90 | 0.00 | 46.90 |
| Low3 | 25.43 | 22.13 | 3.56 | 3.16 | 0.40 | 48.09 |
| Average | 40.97 | 42.95 | 12.12 | 5.14 | 0.40 | 71.28 |

**Table S19. The types hit rate of Attention Weights values low three segments in TN class (75% threshold)**

|  | ***Domain* (%)** | ***Region* (%)** | ***Compositional bias* (%)** | ***Repeat* (%)** | ***Motif* (%)** | **All (%)** |
| --- | --- | --- | --- | --- | --- | --- |
| Low1 | 16.07 | 18.45 | 2.64 | 0.40 | 0.00 | 34.26 |
| Low2 | 19.24 | 18.58 | 2.90 | 0.92 | 0.00 | 37.68 |
| Low3 | 19.50 | 16.86 | 1.19 | 1.32 | 0.40 | 37.81 |
| Average | 36.10 | 35.05 | 6.46 | 1.98 | 0.40 | 62.58 |

**Table S20. The types hit rate of Attention Weights values high top three segments in FP class (25% threshold)**

|  | ***Domain* (%)** | ***Region* (%)** | ***Compositional bias* (%)** | ***Repeat* (%)** | ***Motif* (%)** | **All (%)** |
| --- | --- | --- | --- | --- | --- | --- |
| Top1 | 37.95 | 28.35 | 5.80 | 5.36 | 0.45 | 63.62 |
| Top2 | 34.82 | 34.60 | 9.15 | 3.79 | 0.45 | 67.63 |
| Top3 | 27.90 | 39.06 | 12.72 | 4.24 | 0.89 | 66.07 |
| Average | 55.58 | 61.38 | 23.88 | 8.04 | 1.79 | 85.49 |

**Table S21. The types hit rate of Attention Weights values high top three segments in FP class (50% threshold)**

|  | ***Domain* (%)** | ***Region* (%)** | ***Compositional bias* (%)** | ***Repeat* (%)** | ***Motif* (%)** | **All (%)** |
| --- | --- | --- | --- | --- | --- | --- |
| Top1 | 36.16 | 24.33 | 3.57 | 4.91 | 0.22 | 59.82 |
| Top2 | 33.48 | 29.46 | 4.46 | 3.35 | 0.00 | 62.50 |
| Top3 | 25.00 | 31.47 | 6.47 | 3.57 | 0.22 | 57.59 |
| Average | 54.46 | 53.35 | 12.95 | 7.14 | 0.45 | 81.70 |

**Table S22. The types hit rate of Attention Weights values high top three segments in FP class (75% threshold)**

|  | ***Domain* (%)** | ***Region* (%)** | ***Compositional bias* (%)** | ***Repeat* (%)** | ***Motif* (%)** | **All (%)** |
| --- | --- | --- | --- | --- | --- | --- |
| Top1 | 33.26 | 21.88 | 2.01 | 4.69 | 0.00 | 55.58 |
| Top2 | 30.58 | 23.44 | 1.56 | 2.68 | 0.00 | 54.46 |
| Top3 | 21.88 | 24.55 | 3.79 | 3.35 | 0.22 | 48.66 |
| Average | 50.89 | 45.76 | 6.47 | 7.14 | 0.22 | 77.68 |

**Table S23. The types hit rate of Attention Weights values low three segments in FP class (25% threshold)**

|  | ***Domain* (%)** | ***Region* (%)** | ***Compositional bias* (%)** | ***Repeat* (%)** | ***Motif* (%)** | **All (%)** |
| --- | --- | --- | --- | --- | --- | --- |
| Low1 | 39.29 | 28.57 | 5.58 | 4.91 | 1.56 | 62.95 |
| Low2 | 39.73 | 27.01 | 6.25 | 4.24 | 1.12 | 62.50 |
| Low3 | 39.73 | 31.03 | 7.37 | 4.69 | 0.89 | 66.07 |
| Average | 58.48 | 50.67 | 14.96 | 8.93 | 2.46 | 84.60 |

**Table S24. The types hit rate of Attention Weights values low three segments in FP class (50% threshold)**

|  | ***Domain* (%)** | ***Region* (%)** | ***Compositional bias* (%)** | ***Repeat* (%)** | ***Motif* (%)** | **All (%)** |
| --- | --- | --- | --- | --- | --- | --- |
| Low1 | 33.26 | 21.43 | 2.01 | 4.46 | 0.22 | 54.24 |
| Low2 | 35.27 | 20.54 | 2.90 | 3.35 | 0.45 | 54.91 |
| Low3 | 35.94 | 24.11 | 3.79 | 3.79 | 0.22 | 58.93 |
| Average | 54.24 | 39.96 | 6.70 | 7.81 | 0.89 | 78.57 |

**Table S25. The types hit rate of Attention Weights values low three segments in FP class (75% threshold**

|  | ***Domain* (%)** | ***Region* (%)** | ***Compositional bias* (%)** | ***Repeat* (%)** | ***Motif* (%)** | **All (%)** |
| --- | --- | --- | --- | --- | --- | --- |
| Low1 | 26.12 | 15.40 | 1.79 | 2.46 | 0.22 | 43.53 |
| Low2 | 28.79 | 15.40 | 2.01 | 2.68 | 0.22 | 46.43 |
| Low3 | 30.36 | 18.53 | 2.23 | 2.90 | 0.22 | 51.34 |
| Average | 47.10 | 30.80 | 4.46 | 5.36 | 0.67 | 70.98 |

**Table S26. The types hit rate of Attention Weights values high top three segments in FN class (25% threshold)**

|  | ***Domain* (%)** | ***Region* (%)** | ***Compositional bias* (%)** | ***Repeat* (%)** | ***Motif* (%)** | **All (%)** |
| --- | --- | --- | --- | --- | --- | --- |
| Top1 | 21.98 | 29.67 | 10.99 | 0.00 | 0.00 | 50.55 |
| Top2 | 14.29 | 31.87 | 9.89 | 7.69 | 0.00 | 48.35 |
| Top3 | 21.98 | 25.27 | 6.59 | 12.09 | 0.00 | 52.75 |
| Average | 32.97 | 59.34 | 25.27 | 16.48 | 0.00 | 73.63 |

**Table S27. The types hit rate of Attention Weights values high top three segments in FN class (50% threshold)**

|  | ***Domain* (%)** | ***Region* (%)** | ***Compositional bias* (%)** | ***Repeat* (%)** | ***Motif* (%)** | **All (%)** |
| --- | --- | --- | --- | --- | --- | --- |
| Top1 | 20.88 | 25.27 | 7.69 | 0.00 | 0.00 | 45.05 |
| Top2 | 14.29 | 26.37 | 5.49 | 5.49 | 0.00 | 43.96 |
| Top3 | 21.98 | 19.78 | 3.30 | 9.89 | 0.00 | 47.25 |
| Average | 31.87 | 49.45 | 15.38 | 13.19 | 0.00 | 72.53 |

**Table S28. The types hit rate of Attention Weights values high top three segments in FN class (75% threshold)**

|  | ***Domain* (%)** | ***Region* (%)** | ***Compositional bias* (%)** | ***Repeat* (%)** | ***Motif* (%)** | **All (%)** |
| --- | --- | --- | --- | --- | --- | --- |
| Top1 | 16.48 | 23.08 | 0.00 | 0.00 | 0.00 | 38.46 |
| Top2 | 12.09 | 20.88 | 1.10 | 4.40 | 0.00 | 36.26 |
| Top3 | 19.78 | 14.29 | 1.10 | 9.89 | 0.00 | 40.66 |
| Average | 27.47 | 39.56 | 2.20 | 12.09 | 0.00 | 64.84 |

**Table S29. The types hit rate of Attention Weights values low three segments in FN class (25% threshold)**

|  | ***Domain* (%)** | ***Region* (%)** | ***Compositional bias* (%)** | ***Repeat* (%)** | ***Motif* (%)** | **All (%)** |
| --- | --- | --- | --- | --- | --- | --- |
| Low1 | 10.99 | 20.88 | 4.40 | 6.59 | 0.00 | 31.87 |
| Low2 | 15.38 | 24.18 | 2.20 | 6.59 | 0.00 | 40.66 |
| Low3 | 8.79 | 20.88 | 5.49 | 10.99 | 0.00 | 35.16 |
| Average | 19.78 | 35.16 | 8.79 | 17.58 | 0.00 | 50.55 |

**Table S30. The types hit rate of Attention Weights values low three segments in FN class (50% threshold)**

|  | ***Domain* (%)** | ***Region* (%)** | ***Compositional bias* (%)** | ***Repeat* (%)** | ***Motif* (%)** | **All (%)** |
| --- | --- | --- | --- | --- | --- | --- |
| Low1 | 9.89 | 15.38 | 0.00 | 5.49 | 0.00 | 26.37 |
| Low2 | 13.19 | 20.88 | 1.10 | 6.59 | 0.00 | 37.26 |
| Low3 | 5.49 | 17.58 | 3.30 | 7.69 | 0.00 | 28.57 |
| Average | 17.58 | 28.57 | 4.40 | 13.19 | 0.00 | 46.15 |

**Table S31. The types hit rate of Attention Weights values low three segments in FN class (75% threshold)**

|  | ***Domain* (%)** | ***Region* (%)** | ***Compositional bias* (%)** | ***Repeat* (%)** | ***Motif* (%)** | **All (%)** |
| --- | --- | --- | --- | --- | --- | --- |
| Low1 | 7.69 | 13.19 | 0.00 | 4.40 | 0.00 | 25.27 |
| Low2 | 9.89 | 15.38 | 0.00 | 5.49 | 0.00 | 27.47 |
| Low3 | 4.40 | 14.29 | 1.10 | 4.40 | 0.00 | 20.88 |
| Average | 12.09 | 24.18 | 1.10 | 8.79 | 0.00 | 40.66 |
